# Standing at the Gateway to Europe - The Genetic Structure of Western Balkan Populations Based on Autosomal and Haploid Markers

**DOI:** 10.1371/journal.pone.0105090

**Published:** 2014-08-22

**Authors:** Lejla Kovacevic, Kristiina Tambets, Anne-Mai Ilumäe, Alena Kushniarevich, Bayazit Yunusbayev, Anu Solnik, Tamer Bego, Dragan Primorac, Vedrana Skaro, Andreja Leskovac, Zlatko Jakovski, Katja Drobnic, Helle-Viivi Tolk, Sandra Kovacevic, Pavao Rudan, Ene Metspalu, Damir Marjanovic

**Affiliations:** 1 Estonian Biocentre and Department of Evolutionary Biology, University of Tartu, Estonia; 2 Institute for Genetic Engineering and Biotechnology, Sarajevo, Bosnia and Herzegovina; 3 Institute of Biochemistry and Genetics, Ufa Research Centre, RAS, Ufa, Bashkortostan, Russia; 4 Faculty of Pharmacy, University of Sarajevo; Bosnia and Herzegovina; 5 University Center of Forensic Science, Split, Croatia; 6 Genos doo, Zagreb, Croatia; 7 Vinca Institute of Nuclear Sciences, University of Belgrade, Belgrade, Serbia; 8 Institute for forensic medicine, criminology and and medical deontology, Medical Faculty, University of St. Cyril and Methodius, Skopje, F.Y.R of Macedonia; 9 National forensic laboratory, Ministry of the Interior, Slovenia; 10 Forensic Center, Police Directorate, Danilovgrad, Montenegro; 11 Croatian Academy of Science and Art, Zagreb, Croatia; Democritus University of Thrace, Greece

## Abstract

Contemporary inhabitants of the Balkan Peninsula belong to several ethnic groups of diverse cultural background. In this study, three ethnic groups from Bosnia and Herzegovina - Bosniacs, Bosnian Croats and Bosnian Serbs - as well as the populations of Serbians, Croatians, Macedonians from the former Yugoslav Republic of Macedonia, Montenegrins and Kosovars have been characterized for the genetic variation of 660 000 genome-wide autosomal single nucleotide polymorphisms and for haploid markers. New autosomal data of the 70 individuals together with previously published data of 20 individuals from the populations of the Western Balkan region in a context of 695 samples of global range have been analysed. Comparison of the variation data of autosomal and haploid lineages of the studied Western Balkan populations reveals a concordance of the data in both sets and the genetic uniformity of the studied populations, especially of Western South-Slavic speakers. The genetic variation of Western Balkan populations reveals the continuity between the Middle East and Europe via the Balkan region and supports the scenario that one of the major routes of ancient gene flows and admixture went through the Balkan Peninsula.

## Introduction

The Balkan Peninsula has been continuously settled by anatomically modern humans (AMH) since the Upper Paleolithic era [1]–[4]. The rich archaeological heritage of the region from the period of transition between Middle and Upper Paleolithics in Europe and the traces of different technologies from traditionally Neanderthal associated Mousterian to Ceramic industries of Neolithics [5]–[10] shows the importance of the area for understanding the spread of AMH across the continent [6],[11]. This region has been a probable gateway to Europe for first settlers [12], [13], as well as one of the refugial areas during the Last Glacial Maximum (LGM) [14], [15]. The process of the peopling of the Western Balkans – a crossroad for people moving in different times to and from Europe and beyond - was extensively shaped by several historical episodes. The transition of hunting-gathering to farming in terms of the contrasting influence of pioneering agriculturalists from Anatolia and Mesolithic foragers in this area was probably complex [16], [17]. At the beginning of the second millennium BC the Balkan region was inhabited by different Illyrian tribes, which established the oldest central-western Balkan civilization [18]. The area was also the birth place of two of the world's greatest civilizations - the ancient Greek and the Byzantine Empire. The split of the Roman Empire in 395 AD divided the region into two parts, with the borderline running from Sirmium in the north (Sremska Mitrovica, Serbia) to Skadar Lake in the south (North Albania) [19]. At the same time, the Balkan region served as a frontier between the civilization of the Empire and the barbarian tribes beyond the Danube, which settled in the Balkan in the late 6^th^ century [20], [21]. The first barbarian conquerors in the Balkans were West Goths in 410 AD [22]. In the 6^th^ century, the Slavs had occupied the northern parts of the Danube basin and continued their way to the south. It is believed that part of the Illyrians was assimilated and the other part was forced to move south - into the territory of present-day Albania [19]. During the Great Migrations, next to the Goths and Slavs, the Mongolian tribes moved from the Central Asiatic Plateau to the Balkan Peninsula. The first of these groups of Eastern nomads to make an appearance in the Balkan were Turkic tribes: the Huns and Eurasian Avars [22], [23]. From the 15^th^ until 19^th^ century the Peninsula was under the Ottoman control [19], [22], [24].

**Figure 1:**
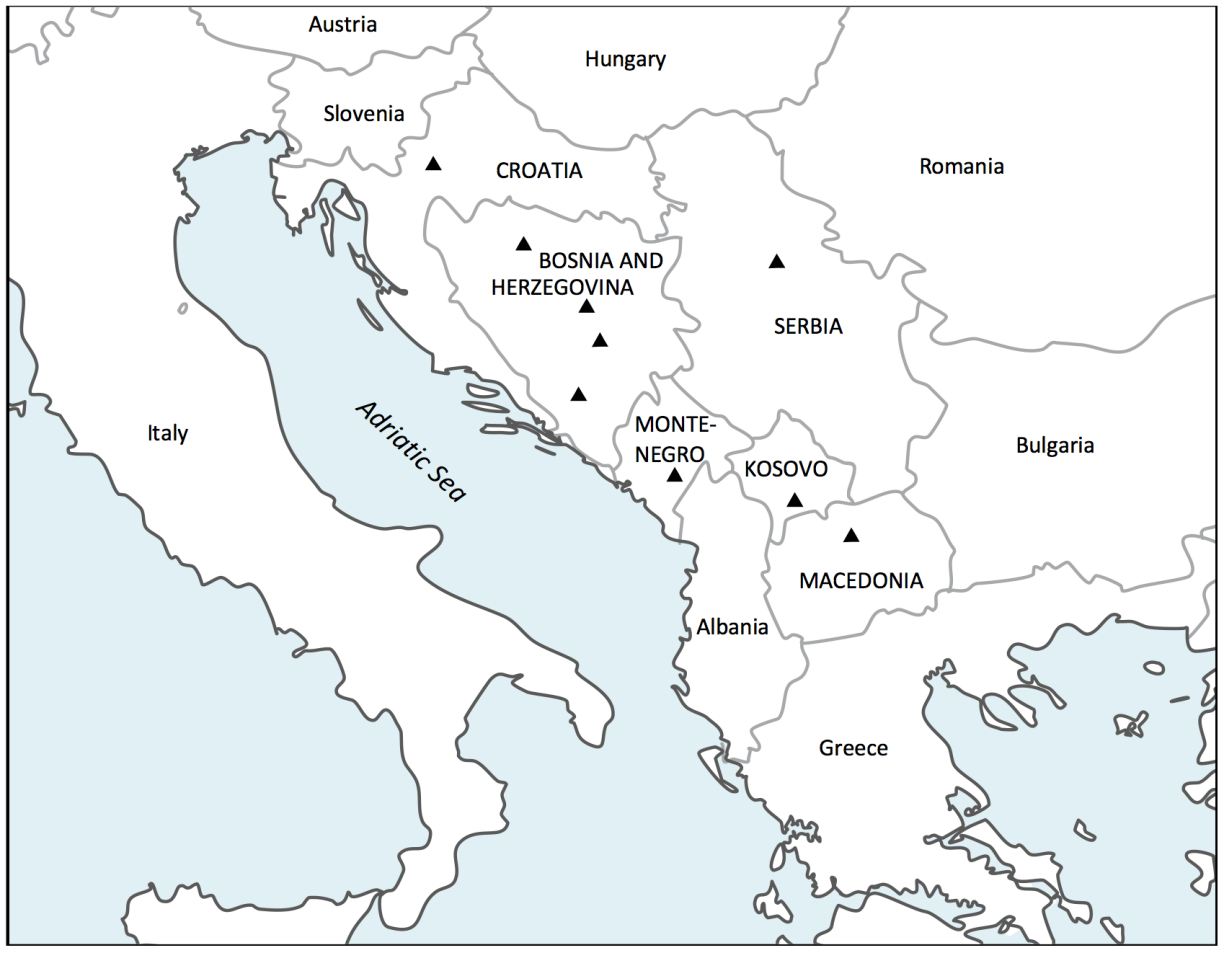
Map of the Western Balkan region with triangles corresponding to the regions from where blood samples were collected. The sample of Bosnia and Herzegovina consisted of subsamples of three main ethnic groups: Bosniacs (Sarajevo and Zavidovici), Bosnian Croats (Central Bosnia - Zepce and Maglaj; South Bosnia and Herzegovina - Mostar, Grude, Livno, Capljina), Bosnian Serbs (Doboj and Banjaluka region); Croatia (mainland, Zagreb region), Serbia (Belgrade region), Montenegro (Podgorica), Kosovo (Pristina and Prizren) and Macedonia (Skopje).

Today, the Western Balkan territory (Figure 1) is inhabited by several ethnic groups of multi-religious and linguistic backgrounds. Ethnicity typically emphasizes linguistic, cultural, religious, as well as political aspects, which are human group specific, and are sometimes interpreted in different ways [25]. In this context, the term refers to religious and linguistic identity. All these groups were encompassed by the countries of the former Yugoslavian Federation and share a common recent history until 1991/1992 when a political conflict resulted in the disintegration of the Federation.

During the last two decades the variation of uniparentally inherited markers such as mitochondrial DNA (mtDNA) and the non-recombining part of Y chromosome (NRY) have been exploited in population genetic studies in order to disentangle the problems of the diversity and dispersal of humans both in global and local contexts [26]–[28]. Recently, Western Balkan populations have been studied intensively from the uniparental perspective [17], [29]–[34]. Genetic analysis based upon the variation of Y chromosome haplogroups (hgs) has revealed that the populations of Western Balkan countries share a large fraction of the ancient gene pool of Southeastern Europe, where 70% of the paternal lineages consist of five European-specific hgs: E3b1, I-P37(xM26), J2, R1a, and R1b [31]. Marjanovic et al. [32] suggested that the frequency of NRY hg I-P37 observed in Bosnia and Herzegovina is particularly high and could be partially attributed to genetic drift. High frequencies of hg I-P37 are observed both in Bosniacs (Bosnian Muslims) (43.5%) and Bosnian Serbs (30.9%). This shows that different ethnic groups in Bosnia and Herzegovina share a large subset of their paternal lineages, affected by a major demographic event, the post-LGM expansion. A population with a high frequency of I-P37 from one of the refuges, located possibly in the Balkans, played a great role in the peopling of Bosnia and Herzegovina and surrounding areas. Similar results were observed for Croatian populations [35].

The study of the variation of mtDNA in the population of Bosnia and Herzegovina has shown - like in case of the variation of NRY - that the majority of detected mtDNA hgs among Bosnians belong to the common West Eurasian gene pool [29]. Also, it revealed that the minor part (2%) of Bosnian mtDNA lineages originate from East Eurasia and Africa. The same study observed that the differences between the Slovenian and Bosnian mtDNA pool, were likely due to two different migration waves to the Balkan Peninsula by different groups of Slavs in Middle Age [36], [37]. However, the sampled Bosnian individuals analyzed in that study were of Serbian and Croatian origin. Cvjetan et al. [30] reported that the frequencies of mtDNA hgs in populations from some countries of the former Yugoslavian Federation - Croatia (coast and mainland), Bosnia and Herzegovina, Serbia and Macedonia, including Macedonian Romani - were in concordance with Western Eurasian data. Only for the populations of small Adriatic island isolates, unusual frequencies of some mtDNA lineages have been reported which are otherwise rare in Europe [38]–[40]. Study of Bosch et al. [33], which included Macedonians of the former Yugoslav Republic of Macedonia, Greeks, Romanians and Albanians, as well as five Aromun populations from different parts of the Balkans, suggested that the diversity of both mtDNA and NRY hgs was similar across the Balkans, except for some Aromun populations. According to these studies, the populations of the Balkan Peninsula have been shown to be genetically homogenous and their uniparentally inherited variation is in concordance with the European genetic continuum. However, it was noted that for the better understanding of the genetic history, different intensity of mobility and migration directions of various populations of southeastern Europe, the variation of maternal lineages in the population cluster consisting of Macedonians of the former Yugoslav Republic of Macedonia, Serbians, Croatians, Herzegovinians and Bosnians should be further resolved by higher mtDNA resolution and deeper statistical analysis of sub-groups [30].

The aim of this study was to characterize, in a larger geographical context, the autosomal gene pool of eight Western Balkan populations from six countries - Bosnia and Herzegovina, Croatia, Serbia, former Yugoslav Republic of Macedonia, Montenegro and Kosovo. All studied samples were characterized also for mtDNA and NRY diversity. One of the main questions we address here is whether the whole genome approach with the accent on the variation of autosomal SNPs is in concordance with the information about genetic affinities of the populations of Western Balkan region, revealed by the studies of uniparental markers.

## Material and Methods

### Samples

Genome-wide autosomal markers of 70 Western Balkan individuals from Bosnia and Herzegovina, Serbia, Montenegro, Kosovo and former Yugoslav Republic of Macedonia (see map in Figure 1) together with the published autosomal data of 20 Croatians were analyzed in the context of 695 samples of global range (see details from Table S1). The sample of Bosnia and Herzegovina (Bosnians) consisted of subsamples of three main ethnic groups: Bosnian Muslims referred to as Bosniacs, Bosnian Croats and Bosnian Serbs. To distinguish between the Serbian and Croatian individuals of the ethnic groups of Bosnia and Herzegovina from those originating from Serbia and Croatia, we have referred to individuals sampled from Bosnia and Herzegovina as Serbs and Croats and those sampled from Serbia and Croatia as Serbians and Croatians. The cultural background of the studied population is presented in Table S2. DNA samples were collected from unrelated and healthy adult individuals of both sexes. The written informed consent of the volunteers was obtained and their ethnicity as well as ancestry over the last three generations was established. Ethical Committee of the Institute for Genetic Engineering and Biotechnology, University in Sarajevo, Bosnia and Herzegovina, has approved this population genetic research. DNA was extracted following the optimized procedures of Miller et al. [41]. All individuals were genotyped and analyzed also for mtDNA and all male samples for NRY variation. All the details of the larger total sample from where the sub-sample for autosomal analysis was extracted, together with the methods used for the analysis of uniparental markers, are characterized in Text S1.

### Analysis of autosomal variation

In order to apply the whole genome approach 70 samples from the Western Balkan populations were genotyped by the use of the 660 000 SNP array (Human 660W-Quad v1.0 DNA Analysis BeadChip Kit, Illumina, Inc.). The genome-wide SNP data generated for this study can be accessed through the data repository of the National Center for Biotechnology Information – Gene Expression Omnibus (NCBI-GEO): dataset nr. GSE59032, http://www.ncbi.nlm.nih.gov/geo/query/acc.cgi?acc=GSE59032

### Genetic clustering analysis

**Figure 2:**
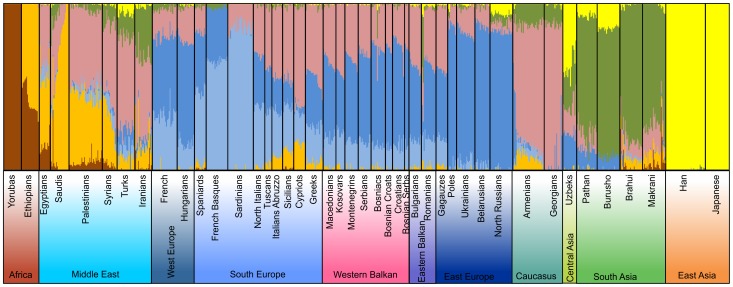
ADMIXTURE analysis of autosomal SNPs of the Western Balkan region in a global context on the resolution level of 7 assumed ancestral populations (See Table S1 for population data).

To investigate the genetic structure of the studied populations, we used a structure-like model-based maximum likelihood algorithm ADMIXTURE [42]. PLINK software v. 1.05 [43] was used to filter the combined data set, in order to include only SNPs of 22 autosomes with minor allele frequency >1% and genotyping success >97%. SNPs in strong linkage disequilibrium (LD, pair-wise genotypic correlation r^2^>0.4) were excluded from the analysis in the window of 200 SNPs (sliding the window by 25 SNPs at a time). The final dataset consisted of 220 727 SNPs and 785 individuals from African, Middle Eastern, Caucasus, European, Central, South and East Asian populations (for details, see Table S1). To monitor convergence between individual runs, we ran ADMIXTURE 100 times at K = 3 to K = 15, the results are presented in Figures 2 and S1.

### Principal Component Analysis and F_ST_

**Figure 3:**
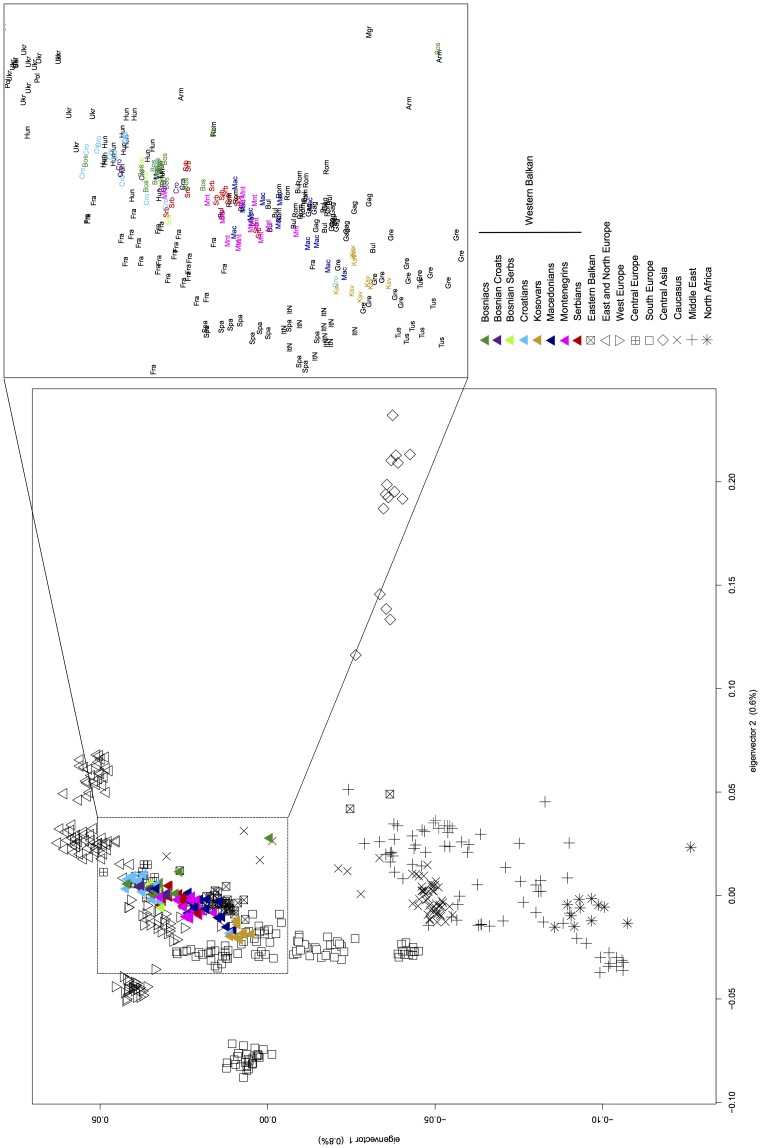
Principal component (PC) analysis of the variation of autosomal SNPs in Western Balkan populations in Eurasian context (PC1 *versus* PC2; see Table S1 for population data).

Dataset for principal component analysis (PCA) was reduced with the exclusion of East and South Asians and Africans, in order to increase the resolution level of the populations from the region of interest (see the details in Table S1, Figure 3). PCA was carried out with the software package SMARTPCA [44], the final dataset after outlier removal consisted of 540 individuals and 200 410 SNPs. All combinations between first five principal components were plotted (Figures S2-S11).

**Figure 4:**
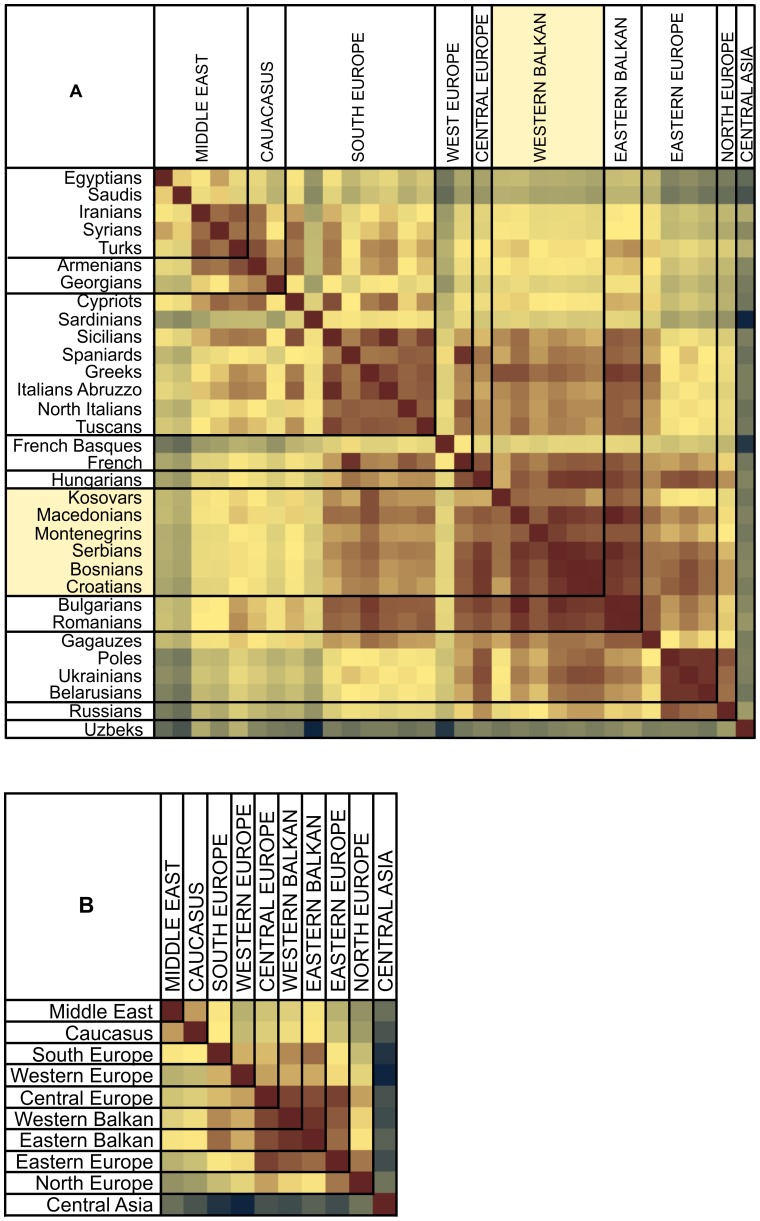
A: F_ST_-distances based on the variation of autosomal SNPs. A: F_ST_-distances of Western Balkans populations in a global context; B: Region-wise F_ST_-distances of the studied populations. F_ST_-values are from 0,03 (dark blue) to 0,00005 (dark brown).

**Figure 5:**
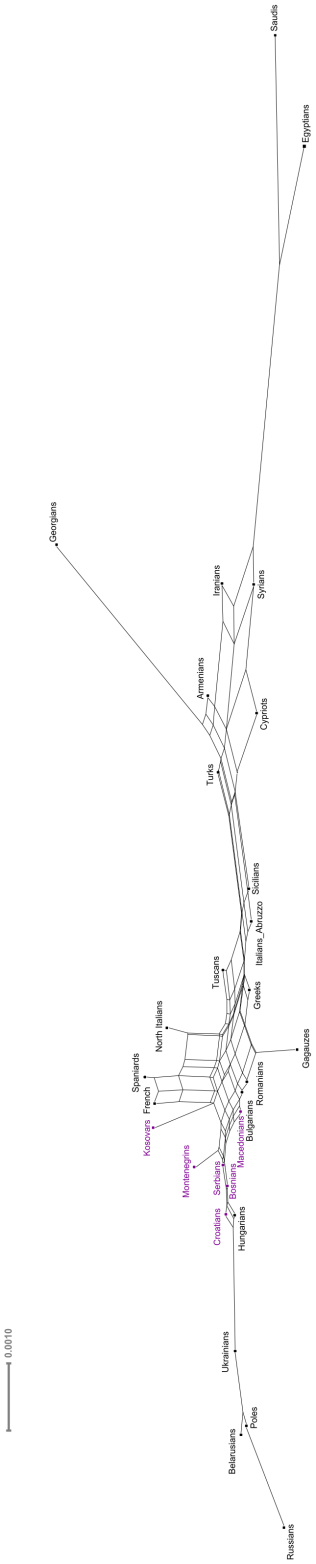
Network of 29 populations constructed with the Neighbor-net approach from F_ST_ distances based on the variation of autosomal SNPs. Western Balkan populations are indicated with violet color.

Pairwise genetic differentiation indices (F_ST_ values) for the same dataset used for PCA were estimated between populations, and regional groups for all autosomal SNPs, using the approach of Weir and Cockerham [45] as in [46]: the total number of populations was 32 and the total number of samples after quality control was 541 (Table S1; Figure 4A,B). A distance matrix of F_ST_ values for the populations specified in Table S1 was used to perform a phylogenetic network analysis (Figure 5) using the Neighbor-net approach [47] and visualized with the EqualAngle method implemented in SplitsTree v4.13.1[48].

### TreeMix

**Figure 6:**
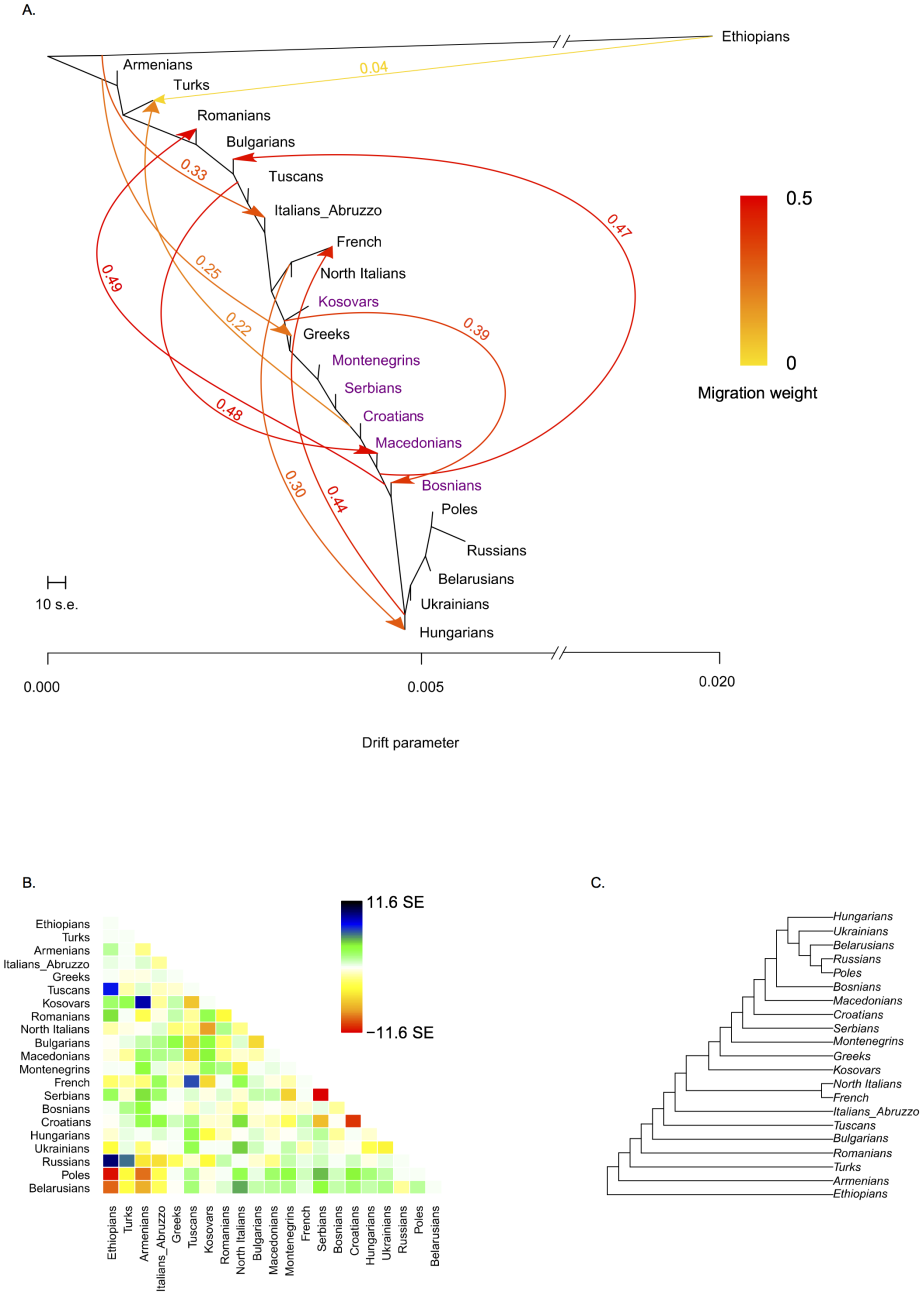
TreeMix analysis of Western Balkan and surrounding populations (see Table S1 for population data). TreeMix graph represents the model of 10 gene-flow events within the sample. A. The population tree with gene-flow (migration) events. The scalebar specifies the weight of a migration, precise value of it is shown on the migration edges; B. Residuals plot; C. Ultrametric tree.

To analyze the population splits and migration events the software TreeMix [49] was used. The dataset (Table S1) consisted of Western and Eastern Balkan populations in the background of a set of South, West and East European populations, the Ethiopians were used as an outgroup. The same filters described above were used, ending up with the dataset of 351 individuals and 202 936 SNPs. We used –k 200 setting to further account for the LD following the TreeMix manual. 100 TreeMix runs for each model of 0 to 10 migration events were performed, the graphs and residual plots were constructed according to the manual using R [50]. At least six best runs arriving at similar log-likelihood (LL) scores for each migration model were examined and all these ended up with very similar LLs and tree topologies. We have chosen to discuss the results with the example of a TreeMix model with the best LL (1371,95), assuming 10 migrations presented in Figure 6. We have also run three population test to calculate a f3-statistic [51], [52] for the same sample set of 21 populations used in the TreeMix analysis for all possible triplets. For this we used the software Threepop within TreeMix package [49]. The total number of SNPs was 202 936 and the f3 of the LD-pruned dataset has been estimated in 1014 blocks. Significant (Z-score is ≤−2) negative values of f3(C; A,B) reflect a signal that population C has arisen from an admixture between groups related to populations A and B. The results are presented in Table S3.

### Analysis of segments identical by descent

The analysis was designed to compare patterns of shared tracts that are identical by descent (*ibd*) between different ethno religious groups of Western Balkan region with Middle Eastern populations. The Ottoman rule over the Balkans during 15–19 cc AD led *inter alia* to the conversion of the local people to Islam, the largest number of whose assumed descendants live in contemporary Bosnia and Kosovo [53]. We questioned whether this cultural transformation was associated with a gene flow between Middle Eastern and Balkan populations. To do so we considered separately the Muslim (Bosniacs, Kosovars) and non-Muslim (Bosnian Croats and Serbs, Croatians, Serbians, Slovenians, Macedonians and Montenegrins) populations of Western Balkan region and calculated pairwise *ibd* sharing for each of these populations and Middle Eastern populations (Turks, Saudis, Palestinians, Iranians, Syrians). The details of the dataset has been characterized in Table S1.

**Figure 7:**
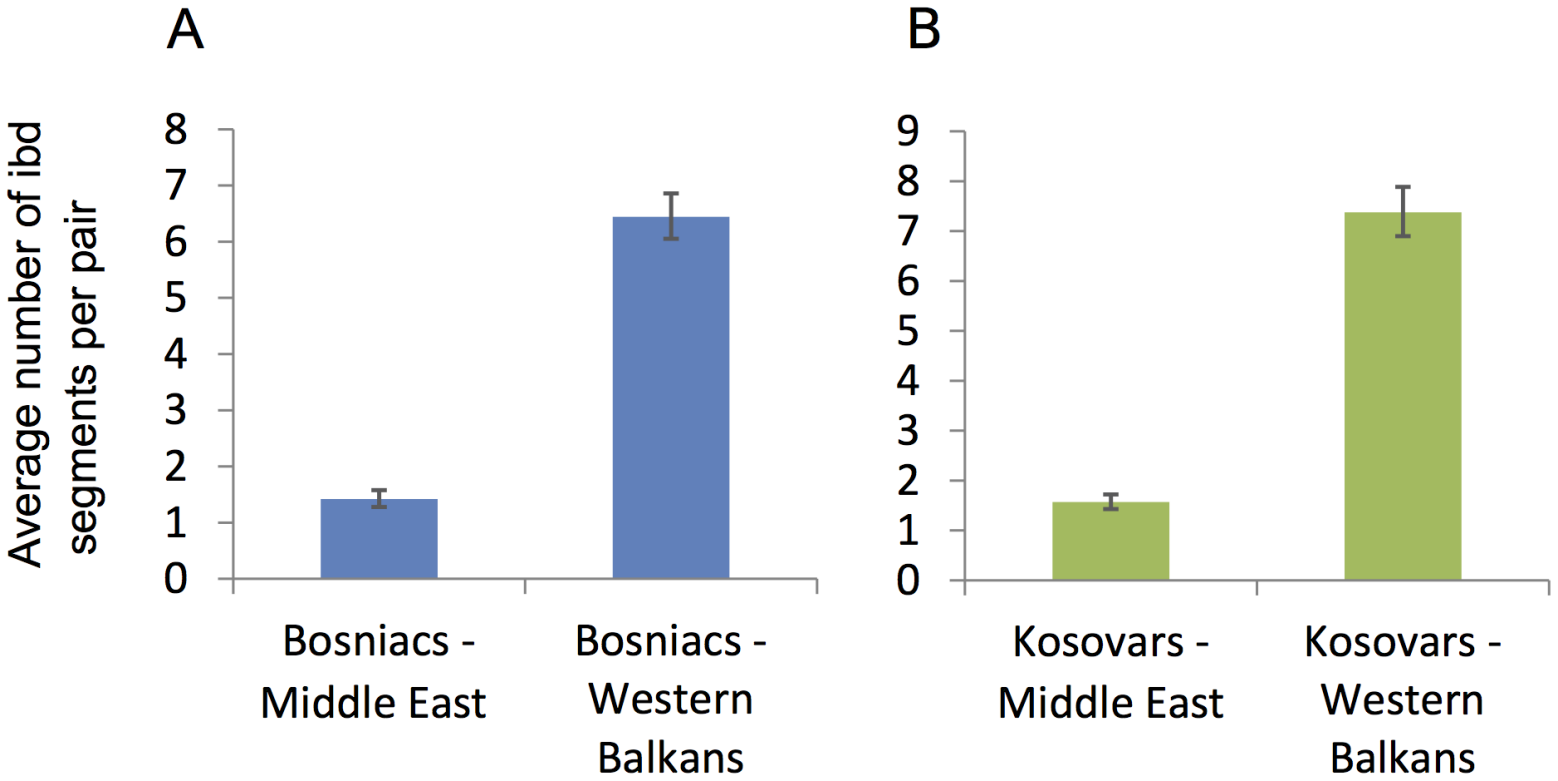
Average number of *ibd* segments per pair shared between Muslim Western Balkan populations (A – Bosniacs; B - Kosovars) and Middle Eastern (Saudis, Iranians, Syrians, Turks, Palestinians) and other non-Muslim Western Balkan populations (Bosnian Croats and Serbs, Croatians, Macedonians, Serbians, Montenegrins).

We used the fastIBD (fIBD) algorithm implemented in BEAGLE software package (http://faculty.washington.edu/browning/beagle/beagle.html) [54] to detect chromosomal segments *ibd* between pairs of individuals. The fIBD algorithm was applied to the 22 autosomes in 10 iterations and the IBD threshold was set to 1e–10. Since the power of the fIBD algorithm to detect segments shorter than 1 centiMorgan (cM) is low, we considered only *ibd* segments longer than 1cM. We summarized *ibd* sharing for six classes of *ibd* segments (1–2 cM, 2–3 cM, 3–4 cM, and 4–5 cM). We estimated an average number of *ibd* segments per pair of individuals for Muslim and non-Muslim populations of Western Balkan *vs* Middle Eastern populations (Figure 7, Table S4). Furthermore, we calculated the average total length of genome shared identical by descent (in cM for four length classes: 1–2, 2–3, 3–4, 4–5 and 5–6) for Muslim Western Balkan populations *vs* each Middle Eastern population for each length class. To test whether observed level of *ibd* sharing between Muslim Western Balkan populations and Middle Eastern populations can be expected by chance, we performed a permutation test. For this, we considered pooled non-Muslim Western Balkan populations as a background and applied the statistical approach described in Yunusbayev et al. [55]. We compared *ibd* sharing from permuted samples to that of Muslim Western Balkan populations and recorded the number of tests showing equal or higher values. The total number of comparable values was divided by total number of permutations to obtain *p*-value (Figure S12).

### Mantel test

The Mantel test (Table 1) with 10 000 permutations for analyzing the correlation between the variation of linguistic, geographical and genetic parameters was conducted by the use of Arlequin software v3.5 [56].

**Table 1:** Correlation analysis between genetic, geographical and linguistic variation. Results of Mantel test.

		Autosomes			MtDNA			Y chromosome	
	Correlation coefficient	P-value	%^2^	Correlation coefficient	P-value	%^2^	Correlation coefficient	P-value	%^2^
Genetics and geography	0,05	0,32	0,00	−0,21	0,69	0,05	0,35	0,08	0,12
Genetics and geography (linguistics held constant)	−0,44	0,92	0,00	−0,34	0,88	0,04	0,31	0,14	0,11
Genetics and linguistics^1^	0,87	0,05	0,75	0,85	0,10	0,72	0,20	0,24	0,04
Genetics and linguistics (geography held constant)	0,89	0,07	0,80	0,86	0,10	0,71	0,10	0,33	0,02
Unexplained genetic variance			0,20			0,25			0,87

1 linguistic affiliations are as in [65].

2 proportion of genetic variation described by given parameter.

## Results and Discussion

### ADMIXTURE analysis of autosomal variation

The analysis of the population structure based on the autosomal variation of the studied Western Balkan populations revealed that their genetic profiles agree well with their geographical position in between the Middle East and the rest of Europe, being closest to the Eastern Balkan and South European populations (Figures 2). The lowest presented level of three ancestral components (K) of ADMIXTURE analysis (K3) separates the African (brown), European (blue) and Asian (yellow) influences in the present gene pool of populations (Figure S1). The African component is absent and the East Asian component can be seen only in trace amounts in Western Balkan populations, but the latter becomes more visible, albeit at low frequencies, in East Slavs/East Europe. K4 brings along the South Asian/Middle Eastern component (green), that at the higher resolution levels (K>5) is left to represent mostly the South Asian populations and its signal in Western Balkan populations is almost not visible. At higher K level the orange Middle Eastern (K>4), light blue European (K>5) and beige Caucasus component (K>6) appear (Figure S1). The most illustrative population structure for the populations of the Western Balkan area is achieved at K = 7 (Figures 2, S1), with three dominant ancestral components. Beside the most apparent dark blue European component, a largely South/West-European-specific light blue and a beige component, shared mostly with the populations from the Caucasus and the Middle East are observed. These two are much more apparent in South Slavic-speaking populations as well as in southern Europeans in general than in North-East Europe including East Slavic speakers, where the dark blue European component is by far the most dominant. The ADMIXTURE profiles of all three ethnic groups of Bosnia are almost identical (Figure 2). The South/West-European component is almost uniformly present in all Western Balkan populations. According to the proportion of different European components at K>6 (Figure S1), the Western Balkan populations have closer genetic affinities with South Europeans than with the geographically more distant West Europeans. The presence of the South/West European light blue component in Eastern-Slavic speakers – Ukrainians, Belarusians and Northwestern Russians is negligible (Figure S1). In Western Balkan region, the Caucasus/Middle Eastern component increases smoothly towards the south and east and is more evident among Macedonians, Kosovars and Montenegrins than in Croatians or in any ethnic group of Bosnia. Its spread most likely illustrates the gene flow from the Middle East to the rest of West Eurasia through the Balkan Peninsula - and further to Western and Eastern Europe, following the decreasing gradient towards the north.

### Principal Component Analysis and F_ST_ distances of autosomal variation

Like the admixture analysis, the PCA and the Fst distances of autosomal data show that there is no clear intra-regional clustering of Western Balkan populations, but rather a geography-based continuity in the gene flow along the north-south axis (Figures 2, 3, 4, 5, S2, S3). The scatterplot of two first principal components (PCs) in Figure 3 is an approximate reflection of the relative geographical distribution of populations – with the South and Southwestern European populations at one and the East Slavic-speaking populations at the other end of the scale of PC2. Heatmap of F_ST_ -s of the studied populations illustrates short genetic distances between geographically nearby populations (Figure 4A) and regional groups (Figure 4B), with some exceptions – like the French Basques and Sardinians, known as genetic isolates [57]. Although very similar to each other (Figure 4A), some genetic differentiation along north-west to south-east direction observed also in ADMIXTURE analysis (Figure 2) is still evident inside the group of Western Balkan. For visualization of F_ST_ distances between populations (Figure 4A) we constructed a graph with the distance-based Neighbor-net method of software SplitsTree for the populations of interest. The resulting network exemplifies genetic affinity between Western Balkan populations that form a bridge between East-European Slavic speakers and populations from Eastern Balkan and the Middle East (Figure 5). The Croatians and Bosnians are more close to East European populations and largely overlap with Hungarians from Central Europe, while Kosovars and Macedonians cluster closer to Eastern Balkan populations and Gagauzes (Figures 3 and 5). Interestingly, the Gagauzes, who geographically locate in East Europe, are more similar to Eastern and Western Balkan populations according to their autosomal profiles (Figure 2, 3 and 5) than to East Europeans. This agrees with the earlier study of the NRY variation suggesting that the Gagauzes descend from northeastern Bulgaria [58]. The Kosovars deviate the most from other Western Balkan populations – note, that among those they have also the biggest similarity to Greeks (Figures 1, 3 and 5). Serbians and Montenegrins have an intermediate position on PCA plot and on Fst –based network among other Western Balkan populations (Figures 3 and 5). The relative position of Western Balkan populations to each other on the PCA plot does not considerably change in any combination of first five PC-s (Figures S2-11).

### TreeMix analysis

In order to reconstruct the demographic history of the populations of Western Balkan region we ran the TreeMix analysis for the same subset of populations used in ADMIXTURE (Figure 6). The topology of the tree (Figure 6C) as well as the direction and weight of the migration events (Figure 6A) were the same for all 6 best runs with the highest maximum likelihood values for the model of 10 migrations. The tree chosen here as the representative of the analysis reflects close relationships between compared populations, and the division into well-defined clades is not observed, except for French and North Italians (Figure 6A,C). The Western Balkan populations take central position on the tree and are surrounded by the Eastern Balkan and South European populations from one and the Eastern Slavic populations together with Poles and Hungarians from the other side. The latter three form the tipmost branch of the tree. The migration events with the highest weight are directed towards the Eastern Balkan populations – to Romanians (migration weight 0.49) and to Bulgarians (weight 0.47), who have received the considerable gene flow from the root of the edge encompassing East Slavic populations, Poles, Hungarians and Bosnians from Western Balkan. Similarly high weight (0.48) is given to a migration directed from the root of the edge between Bulgarians and Tuscans to Macedonians, but also to the migration from the edge (0.39) between Kosovars and Greeks to Bosnians. The considerable gene flow indicated with the weight close to 0.5 (edges with weight >0.5 are defined as tree edges) in case of three discussed here migration events reflects that it would have been almost equally possible for the TreeMix to transform the migrational edge into “tree” and relocate here the Macedonians next to Tuscans and Bulgarians and Bosnians next to Greeks and Kosovars. Part of the Western Balkan populations - Croatians, Macedonians and Bosnians - together with Eastern Slavic speakers, Poles and Hungarians have contributed also to the gene flow towards the Middle East (Turks, migration weight 0.22). Thus, the results of the TreeMix analysis are mostly consistent with the geographical spread of the sampled populations (Figure 6A) and reflects considerable mutual gene flow between neighboring regions, seen also in the other presented here analyses. According to the results of three population test (Table S3), all Western Balkan populations except Kosovars show clear signs of complex demographic history with admixture from groups related to Eastern Balkan, South European and Slavic-speaking populations both from Balkan Peninsula and East Europe. It has been noted that demographic events like population-specific drift can mask the admixture signals [52], which might be the reason for the lack of admixture signal in the case of Kosovars.

### Analysis of segments identical by descent

To assess potential admixture between Western Balkan and Middle Eastern populations during the Ottoman rule (15–19cc AD) we first analyzed the number of *ibd* segments shared per one pair for Western Balkan and Middle Eastern populations. In average, both Muslim (Bosniacs, Kosovars) and non-Muslim (Bosnian Croats and Serbs, Macedonians, Montenegrins, Serbians and Croatians) of the Western Balkan populations share around 1.5 *ibd* segments per pair with the population from the Middle East (Table S4). This is significantly lower than around 7 *ibd* segments per pair that Bosniacs and Kosovars share with other non-Muslim WB populations (Figure 7, Table S4). Next, we inspected the average total length of genome shared identical by descent in cM for four length classes between Muslim and non-Muslim populations of Western Balkan *vs* Middle Eastern populations. We found that all tested Western Balkan populations, irrespective their ethno religious affiliations, demonstrate similar (p = 0.1–0.9) patterns of *ibd* sharing with Middle Eastern populations for shorter classes of *ibd* segments (1–2, 2–3, 3–4 cM). This is slightly higher with Turks, and lower with Saudis, Syrians, Iranians and Palestinians (Figure S12). For longer *ibd* segments only Kosovars have higher *ibd* relatedness with Palestinians (p = 0.0056 for 4–5 cM ibd segments) and only Bosniacs have higher *ibd* sharing with Turks (p = 0.0097 for 5–6 cM ibd segments) (Figure S12). However, taking into account that in general the number of shared *ibd* segments longer than 4 cM detected between Bosniacs, Kosovars and Middle Eastern populations is very low and that higher *ibd* sharing is not seen for other classes of *ibd* segments, we cannot consider the excess of long *ibd* segments between Bosniacs and Turks, and between Kosovars and Palestinians as sufficient evidence of stronger gene flow between Middle Eastern populations and Muslim populations of Western Balkan as compared to non-Muslim Western Balkan populations.

Taken together, analysis of *ibd* segments reveals similar patterns of *ibd* sharing for Muslim and non-Muslim Western Balkan populations with populations of Middle East, providing thereby little support to a gene flow scenario during the conversion to Islam (15–19 cc AD) in the Balkans. Our analysis of *ibd* sharing agrees with other analyses (Figures 2, 3, 5) which indicate higher relatedness for all the Western Balkan populations and Turks as compared to other Middle Eastern populations, most likely due to geographic proximity.

### Variation of haploid markers of Western Balkan populations

The results of the analysis of mtDNA and NRY are presented in Text S2 and in Supplementary Material (Tables S5-S10, Figures S13-S21). The detailed phylogenetic analysis of maternal lineages of studied here Western Balkan populations (see Tables S5 and Figures S14-18, Text S2) revealed their branching patterns, deeply connected with those of other European and Middle Eastern populations. Like in autosomal analysis, we found only some rare genetic variants from our sample that are not common in European populations. We detected one [0,6% (with 95% credible region (CR) width 0,1–3,1%)] maternal lineage of Eastern Eurasian origin from hg D4 in our sample of Montenegrins (Table S5). Lineages of Eastern Eurasian macrohg M, occasionally seen in many European populations [59] has been detected also in Western Balkan area [29], [30], [33], [39]. An equally minor part [1,1% (CR 0,4–4,0%)] of mtDNAs belong to the set of African origin - two samples of hg L1b was found, one from Serbian and the other from Bosnian Croat population (Table S5). The presence of the same haplotype as well as another African lineage L2a3 has been observed in the region, among Bosnians [29] and Croatians from Korcula island [30], respectively. Outside Africa, the African-specific lineages are the most frequent in populations of the Iberian Peninsula and the Near East, which have experienced the strongest influence of African populations during their history [60], [61]. Regardless, the overall frequency of African lineages in Eurasia [62] is the same as in our sample. The Atlantic slave trade through Portugal, which was the principal destination within Europe [60] and/or the trafficking of African children via the markets of the Ottoman Empire to East Europe in the beginning of 17^th^ century [63] could be one of the reasons for the gene flow from the people of African ancestry to the Western Balkan region.

The number of studied mtDNA samples could not yet be classified into specific sub-clades according to present nomenclature (Table S5). This might indicate that the diversity of maternal lineages in this part of the Balkan Peninsula has region-specific characteristics, which are potentially interesting to investigate by further deeper analysis of mtDNA genomes. We have completely sequenced 5 mtDNAs from the minor region-specific twigs of the global mtDNA tree from hgs K1a (2), N1a (1) and R0a (2) of our sample (Figures S19-21, Text S2). While the sequence variants or their close relatives belonging to the latter two hgs can be found at low frequencies in a wider area of Europe and Middle East [64], this particular sub-branch of hg K1a we found seems to be resticted only to Western Balkan region. We performed a phylogeographic study encompassing 253 samples from the DNA sample collection of Estonian Biocentre, known to belong to hg K1a, but not analyzed at the K1a sub-branch level. These samples were extracted from the set of populations of European, Caucasian and Middle Eastern origin (N = 6488). Six out of 253 K1a samples turned to have transition from T to C at nucleotide position (np) 8870, diagnostic to hg K1a13a [64], all of these were from Croatian mainland sample (N = 440). Two Croatian samples with different HVS-1 motifs were sequenced completely (Croatia.m.(S)199 and Croatia.m.(S)341 in Figure S19). We suggest here to amend the present mtDNA classification with a new sub-clade in hg K1a - K1a13a1, defined by transition from C to T at np 11236 and T to C at np 16093. This new branch of K1a13 encompasses now next to reported GenBank mtDNA (with accession no. JN202723) of Croatian origin also mtDNAs of two individuals from Bosnia and Herzegovina from our sample and two additional mitogenomes from Croatia (Figure S19).

To compare the autosomal results with those of uniparentally inherited markers in Western Balkan region we made a PCA for both mtDNA and Y chromosomal data in a context of selected surrounding populations (Figure S13, see also Text S2). Due to a small sample size of each individual population we pooled the dataset of Western Balkan population together for PCA of mtDNA and NRY data (Figure S13A and B; the results for each Western Balkan population are shown on Figure S13C and D). Here, the Western Balkan populations are closest to their Slavic-speaking neighbours both according to maternal (Czechs and Belarusians, Figure S13A) and paternal (Slovaks, Figure S13B) variation, but it has to be noted that the pooled sample is biased towards northern populations of Western Balkan (Bosnia and Herzegovina, Croatia) and thus represents mostly the variation of this part of the study region. In autosomal analysis, the Bosnians and Croatians are closest to Hungarians, the East Europeans and Eastern Balkan populations are at the same distance from these Western Balkan populations (Figures 3 and S2, S3). East European Slavic-speakers are similar to our pooled Western Balkan sample of PCA also in mtDNA and NRY analyses (Figure S13A and B) and the Hungarians in NRY analysis (Figure S13B). The variation pattern of maternal lineages of the Eastern Balkan populations and Greeks, the most similar populations to southernmost Western Balkan populations (Kosovars, Macedonians, Montenegrins) in autosomal analyses (Figures 2 and 3), are with this sample set more close with mtDNA variation of Central European populations, Austrians and Hungarians (Figure S13A). However, the variation pattern of paternal lineages of Greeks brings them closer to Western Balkan populations, notably also to Macedonian Greeks (Figure S13B). Altogether, the results of the PCA of uniparentally inherited markers, like those of autosomal analysis, reflect mostly the importance of geographical factors on the genetic variation of the region.

### Kosovars – non-Slavic speakers of the Western Balkan region

Compared to the rest of the Western Balkan populations, the Kosovars have a somewhat different cultural and demographic background. All studied Western Balkan populations, except Kosovars, belong to the South Slavic branch of the Indo-European (IE) language family [65] (Table S2). The language spoken by Kosovars, who are sometimes considered to be the descendants of ancient Illyrians [66], belong to the IE family's Albanian branch. Historical linguists have not resolved the position of the Albanian group and the recent results of Gray et al. [67] clearly reflect this uncertainty. It is also important to mention here that historically the traditional social grouping among the Albanians of Kosovo has been a clan. A clan was based on blood related families only through the male line. The clans were exogamous, which means that the brides were aquired from other clans [68]. In certain cases some sub-clans of the large clan considered their supposed common ancestor sufficiently distant in time for them to exchange brides with one another [53]. In many autosomal analyses (see Figures 2, 3, 6, but see also Figure 5) the Kosovars show the closest affinities among Western Balkan populations to Greeks and other South European populations. In our *ibd* analysis, we also did not find evidences for specific gene flow from the Middle East to Kosovars, compared to non-Muslim populations of Western Balkan (Figure 7). However, three population test did not show significant admixture signals for Kosovars and neighboring populations (Table S3), suggesting a different demographic history, most probably a population-specific bottleneck, masking the admixture signal, compared to other Western Balkan populations. We made a correlation analysis between genetic variation and geography/linguistics of all three studied marker sets within the Western Balkan region (Table 1, Text S1). The correlation indexes of autosomes and mtDNA show high, but similarly to NRY insignificant values for the correlation between genetics and linguistics. If the linguistic differences in this dataset are also observed as an indirect indicator of different sociocultural traditions (paternal clans *versus* non-clans) of the Western Balkan populations, the influence of the clan structure to the present genetic variation should be seen the most in the Y chromosomal gene pool of the studied populations – this, however, is not the case. To conclude: the linguistic or religious differences seem to have had no impact on the present variation of uniparentally inherited or autosomal markers in a region.

## Conclusions

We have analyzed and present here the new data of genome-wide autosomal diversity of five Western Balkan populations. The variation analysis of 660K autosomal SNPs of 70 individuals from Western Balkan populations revealed that the genetic uniformity that has been shown by studies of uniparentally inherited markers of these populations can be seen also at the whole-genome level. Thus, culturally diverse Western Balkan populations are genetically very similar to each other. These results, together with the high-resolution analysis of the variation of mtDNA and NRY, let us to affirm that the genetic profiles of Western Balkan populations resemble that of their closest geographical neighbors, and in the global context are in concordance with the geographical distribution of the studied population.

The major variants of the gene pool of present-day Western Balkan populations have developed from a common source without being influenced by major population-specific bottlenecks. In a more general perspective, our results reflect clear genetic continuity between the Middle Eastern and European populations. It has been suggested recently that the Neolithic migrants from Anatolia took mainly the maritime coastal route and island hopping to reach Europe [69]. The genetic variation of the studied here Western Balkan populations lends credence also to extensive, likely multiple and possibly bidirectional gene flows between the Middle East and Europe, traversing the Balkans.

The autosomal analysis as well as mtDNA and NRY data presented in this study contribute to an existing database and for understanding the origins of the peopling of this part of Europe.

## Supporting Information

Figure S1 **ADMIXTURE plots of autosomal SNPs of Western Balkan region in a global context on the resolution level of 3 to 15 assumed ancestral populations (K).** A. Box and whiskers plot of the cross validation (CV) indexes of all 15×100 runs of ADMIXTURE; B. Log-likelihood (LL) scores of all 15×100 runs of ADMIXTURE. Inset shows the variation in the fractions (5%, 10% and 20%) of runs that reached the highest LL values; C. Bar plot displaying individual ancestry estimates for studied populations.
(TIF)

Figure S2 **Principal component (PC) analysis (PC1** ***versus*** **PC2) of the variation of autosomal SNPs in Western Balkan populations (highlighted) in Eurasian context (see Table S1 for population data and abbreviations).**
(TIF)

Figure S3 **Principal component (PC) analysis (PC1** ***versus*** **PC3) of the variation of autosomal SNPs in Western Balkan populations (highlighted) in Eurasian context (see Table S1 for population data and abbreviations).**
(TIF)

Figure S4 **Principal component (PC) analysis (PC1** ***versus*** **PC4) of the variation of autosomal SNPs in Western Balkan populations (highlighted) in Eurasian context (see Table S1 for population data and abbreviations).**
(TIFF)

Figure S5 **Principal component (PC) analysis (PC1** ***versus*** **PC5) of the variation of autosomal SNPs in Western Balkan populations (highlighted) in Eurasian context (see Table S1 for population data and abbreviations).**
(TIF)

Figure S6 **Principal component (PC) analysis (PC2** ***versus*** **PC3) of the variation of autosomal SNPs in Western Balkan populations (highlighted) in Eurasian context (see Table S1 for population data and abbreviations).**
(TIF)

Figure S7 **Principal component (PC) analysis (PC2** ***versus*** **PC4) of the variation of autosomal SNPs in Western Balkan populations (highlighted) in Eurasian context (see Table S1 for population data and abbreviations).**
(TIF)

Figure S8 **Principal component (PC) analysis (PC2** ***versus*** **PC5) of the variation of autosomal SNPs in Western Balkan populations (highlighted) in Eurasian context (see Table S1 for population data and abbreviations).**
(TIF)

Figure S9 **Principal component (PC) analysis (PC3** ***versus*** **PC4) of the variation of autosomal SNPs in Western Balkan populations (highlighted) in Eurasian context (see Table S1 for population data and abbreviations).**
(TIF)

Figure S10 **Principal component (PC) analysis (PC3** ***versus*** **PC5) of the variation of autosomal SNPs in Western Balkan populations (highlighted) in Eurasian context (see Table S1 for population data and abbreviations).**
(TIF)

Figure S11 **Principal component (PC) analysis (PC4** ***versus*** **PC5) of the variation of autosomal SNPs in Western Balkan populations (highlighted) in Eurasian context (see Table S1 for population data and abbreviations).**
(TIF)

Figure S12 **Principal component (PC) analysis based on the frequencies of mtDNA (panels A and C) and NRY haplogroups (panels B and D) of Western Balkan (WB) populations in a context of selected Central and South Europeans and Iranians from the Middle East (see the details of the dataset from Text S1).** A: Pooled mtDNA data of WB populations. PC1 encompasses 36,6% and PC2 20,5% of total mtDNA variation; C: mtDNA data of each WB population plotted separately. PC1 encompasses 18,3% and PC2 16,3% of total mtDNA variation; B: Pooled NRY data of WB populations. PC1 encompasses 32,2% and PC2 24,4% of total NRY variation. D: NRY data of each WB population plotted separately. PC1 encompasses 28,3%, PC2 21,4% of total NRY variation. Abbreviations for studied WB populations are presented in Table S2, abbreviations for populations used for comparison are given in alphabetical order as follows: AUST - Austrians; BEL – Belarusians; BUL – Bulgarians; HUNG - Hungarians from Budapest; CZEC - Czechs; IR - Iranians; MAC.GRK - Macedonian Greeks; N. GRK - Greeks from North Greece; S.IT. - Italians from South Italy; N-E.IT - North-East Italians; ROM – Romanians; SLVK – Slovaks. Symbols on panels indicate geographical origin of populations as follows: triangles - Western Balkan; full circles - Central and East Europe; rhomboids - South Europe and Eastern Balkan; squares - Middle East. The references below are given in Text S1. The obtained NRY data were analyzed jointly with previously published data of 84 Bosniacs, 90 Bosnian Croats, 81 Bosnian Serbs, 118 Croatians, 64 Macedonian Albanians (FYROM Albanians pooled with Macedonians from FYROM) and 55 Albanians from Battaglia et al. 2009 [17], pooled with Kosovars, and 113 Serbians from Pericic et al. 2005 [12], pooled with Serbians and Montenegrins of this study.
(TIF)

Figure S13 **Average total length of genome shared** ***identical by descent*** **between Bosniacs, Kosovars and Near Eastern populations.** Panels A-E indicate five length classes of *ibd* segments: 1–2, 2–3, 3–4, 4–5, 5–6 cM, respectively. Bosniacs and Kosovars are tested Muslim populations from Western Balkans; Macedonians, Montenegrins, Bosnian Croats and Serbs, Croatians, Serbians are non-Muslim populations from Western Balkans, used as a background. Red color of the Western Balkan population name and red circle around the symbol of Middle Eastern population indicates significantly higher *ibd* sharing between these populations as compared to non-Muslim background.
(TIF)

Figure S14 **Median-joining network of mtDNA hg H lineages of Western Balkan populations: A: subhg H1 and its sub-branches; B: other subhgs of hg H. A total number of 19 H1 and 49 other H haplotypes are reported.** Numbers on links indicate the mutations: blue color indicates HVS1 and HVS2 mutations, black color coding region mutations. Polymorphic nucleotide sites are numbered according to Reconstructed Sapiens Reference Sequence. Node size is proportional to absolute haplotype frequency, as it is reported in figure legend.
(TIFF)

Figure S15 **Median joining network of mtDNA hg U lineages.** A total number of 34 U haplotypes are presented. A diagnostic mutation A1811G has been added into the network, but not genotyped in the sample. For further details, see the legend of figure S5.
(TIF)

Figure S16 **Median joining network of mtDNA lineages for hgs J and T in the Western Balkan region.** A total number of 24 J and 10 T haplotypes are presented. For further details, see the legend of figure S5.
(TIF)

Figure S17 **Median joining network of mtDNA lineages of hgs HV, V and R0a.** A total number of 11 V, 8 HV and 2 R0a haplotypes are reported.For further details, see the legend of figure S5.
(TIF)

Figure S18 **Median joining network of mtDNA lineages from hgs W, I and N1b.** A total number of 6 W, 1 I and 3 N1b of haplotypes are reported. For further details, see the legend of figure S5.
(TIF)

Figure S19 **Phylogenetic tree of mtDNA K1a13a complete sequences.** Two samples of Bosnian Croats (BHCB15 and BHCHZ20), and two from Croatia [Croatia.m.(S)199 and Croatia.m.(S)34] are sequenced in this study, the others are from Phylotree mtDNA Build 15. The mutations are given relative to the Reconstructed Sapiens Reference Sequence.
(TIF)

Figure S20 **Phylogenetic tree of mtDNA N1a complete sequences.** One sequence of Croatian from Croatia is sequenced in this study, the others are from Phylotree mtDNA Build 15. The mutations are given relative to the Reconstructed Sapiens Reference Sequence.
(TIF)

Figure S21 **Phylogenetic tree of mtDNA R0a2 complete sequences. Macedonian (MAC16) and Croat from Bosnia and Herzegovina (BHCCB19) are sequenced in this study, the others are from Phylotree mtDNA Build 15. The mutations are given relative to the Reconstructed Sapiens Reference Sequence.**
(TIF)

Table S1 **Sample of populations used for autosomal analyses.**
(XLS)

Table S2 **The ethnolinguistic characteristics of studied Western Balkan populations.**
(XLS)

Table S3 **F3-statistic calculated for all possible triplets of f3(C; A, B) of TreeMix dataset.**
(XLSX)

Table S4 **Average number of IBD segment per pair of individuals.**
(XLSX)

Table S5 **MtDNA HVS-1 and HVS-2 haplotypes of analyzed Western Balkan populations relative to Reconstructed Sapiens Reference Sequence.**
(XLSX)

Table S6 **Y chromosome variation in Western Balkan populations.**
(XLSX)

Table S7 **MtDNA gene and nucleotide diversity of analyzed Western Balkan populations.**
(XLSX)

Table S8 **F_st_-distances of mtDNA HVS1 variation between Western Balkan populations.**
(XLSX)

Table S9 **Results of AMOVA and Mantel test based on mtDNA HVS-1 haplotype or haplogroup frequencies.**
(XLSX)

Table S10 **Estimated coalescense time for the most frequent mtDNA haplogroups in studied Western Balkan populations.**
(XLSX)

Text S1 **Description of the sample and methods of the analyses of mtDNA and NRY.**
(DOCX)

Text S2 **Results of the analyses of mtDNA and NRY variation.**
(DOC)
