# Supplementary figures and images for: Standing at the Gateway to Europe - The Genetic Structure of Western Balkan Populations Based on Autosomal and Haploid Markers
Source: PLoS One. 2014 Aug 22;9(8):e105090. doi: 10.1371/journal.pone.0105090 (PMC4141785; doi:10.1371/journal.pone.0105090)

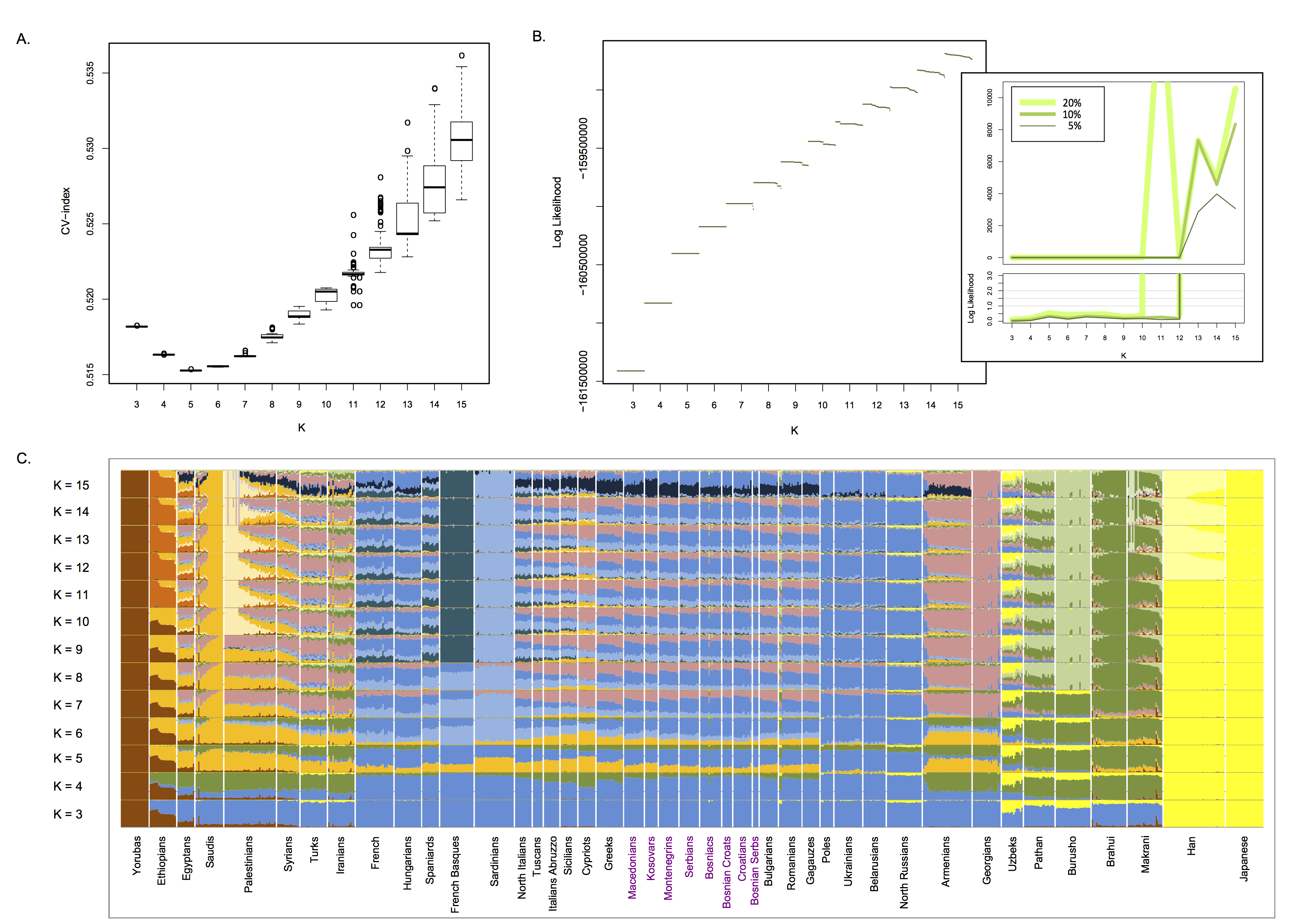

Supplement: Figure S1 — ADMIXTURE plots of autosomal SNPs of Western Balkan region in a global context on the resolution level of 3 to 15 assumed ancestral populations (K). A. Box and whiskers plot of the cross validation (CV) indexes of all 15×100 runs of ADMIXTURE; B. Log-likelihood (LL) scores of all 15×100 runs of ADMIXTURE. Inset shows the variation in the fractions (5%, 10% and 20%) of runs that reached the highest LL values; C. Bar plot displaying individual ancestry estimates for studied populations. (TIF) [file pone.0105090.s001.tif]

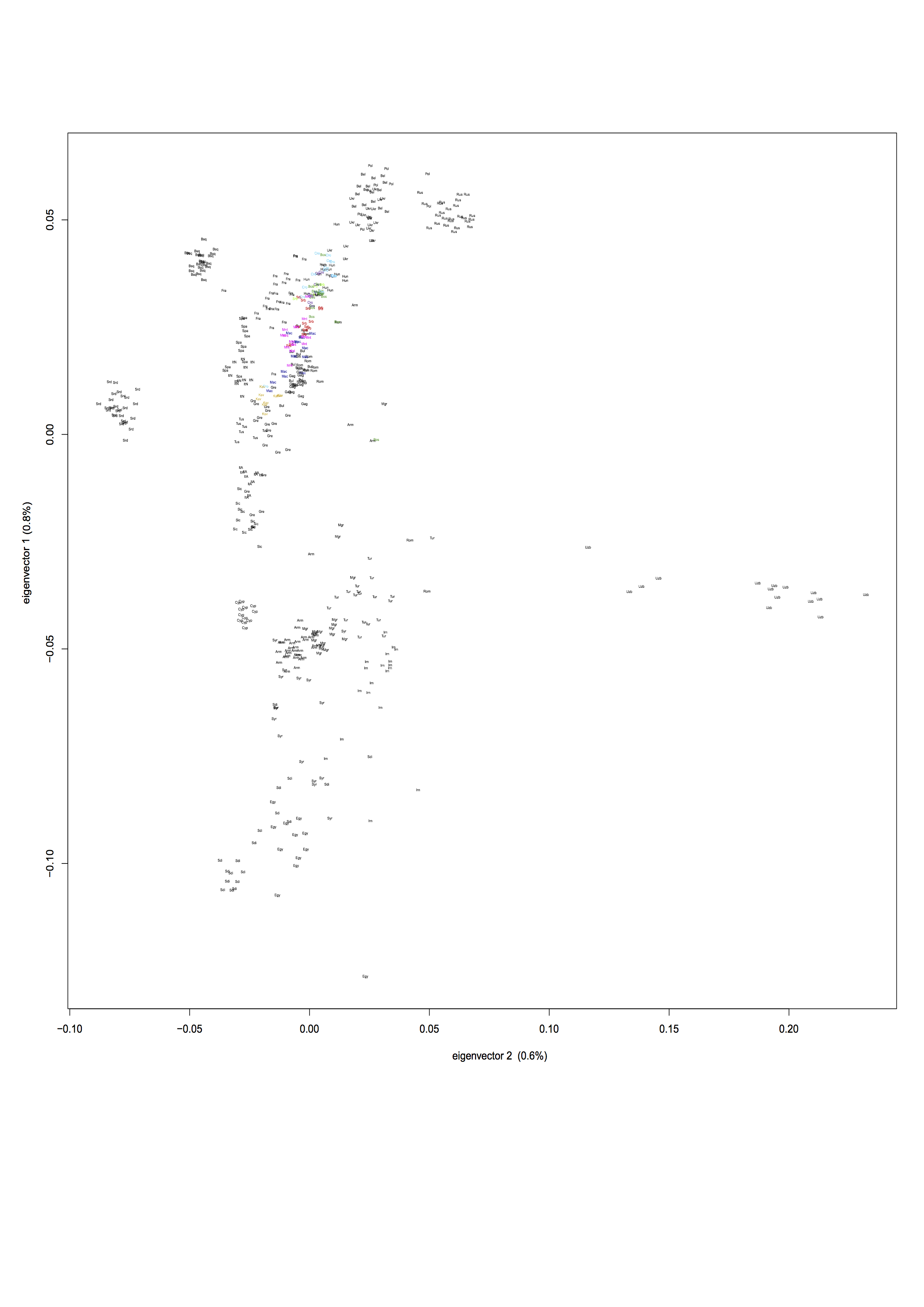

Supplement: Figure S2 — Principal component (PC) analysis (PC1 versus PC2) of the variation of autosomal SNPs in Western Balkan populations (highlighted) in Eurasian context (see Table S1 for population data and abbreviations). (TIF) [file pone.0105090.s002.tif]

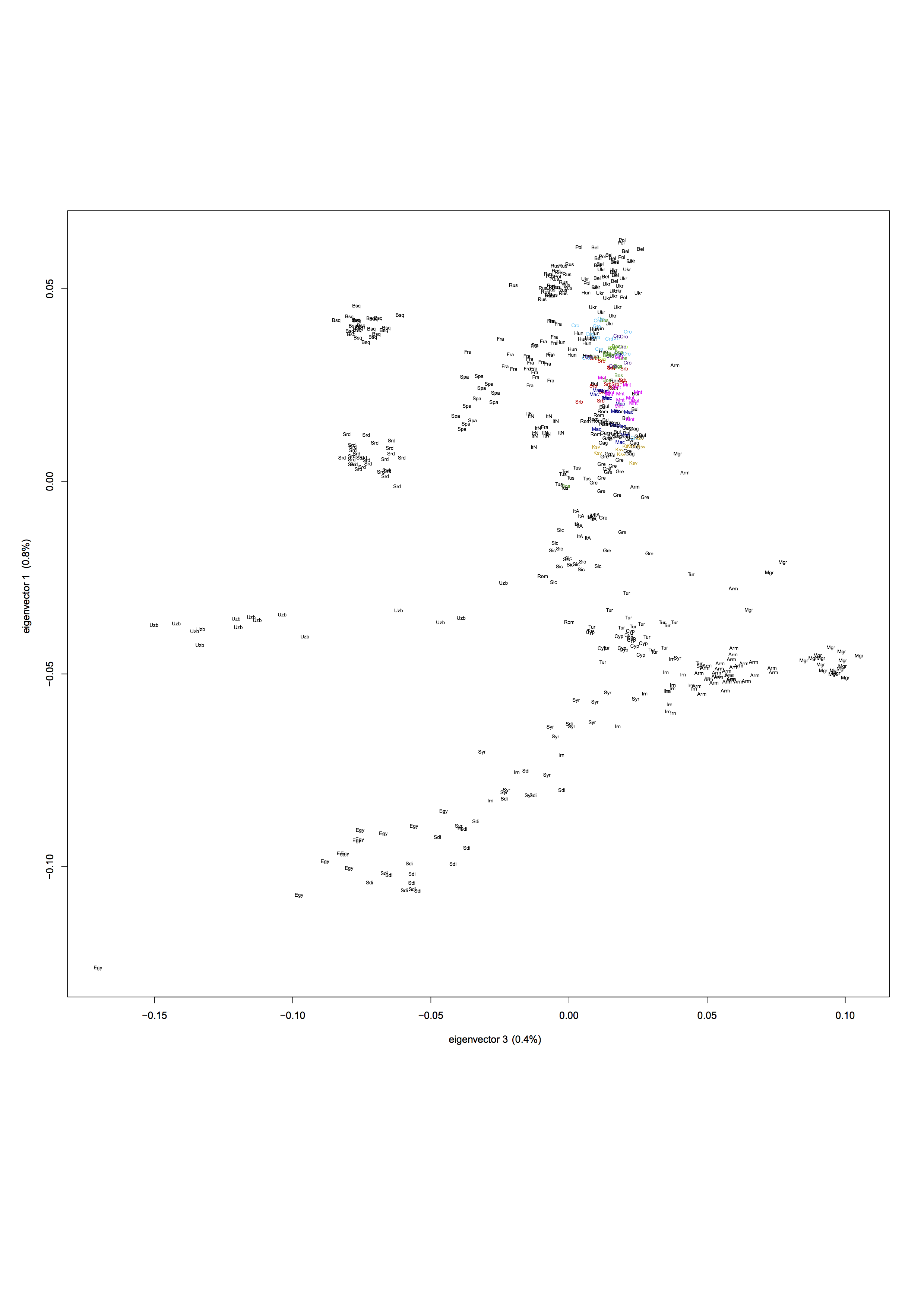

Supplement: Figure S3 — Principal component (PC) analysis (PC1 versus PC3) of the variation of autosomal SNPs in Western Balkan populations (highlighted) in Eurasian context (see Table S1 for population data and abbreviations). (TIF) [file pone.0105090.s003.tif]

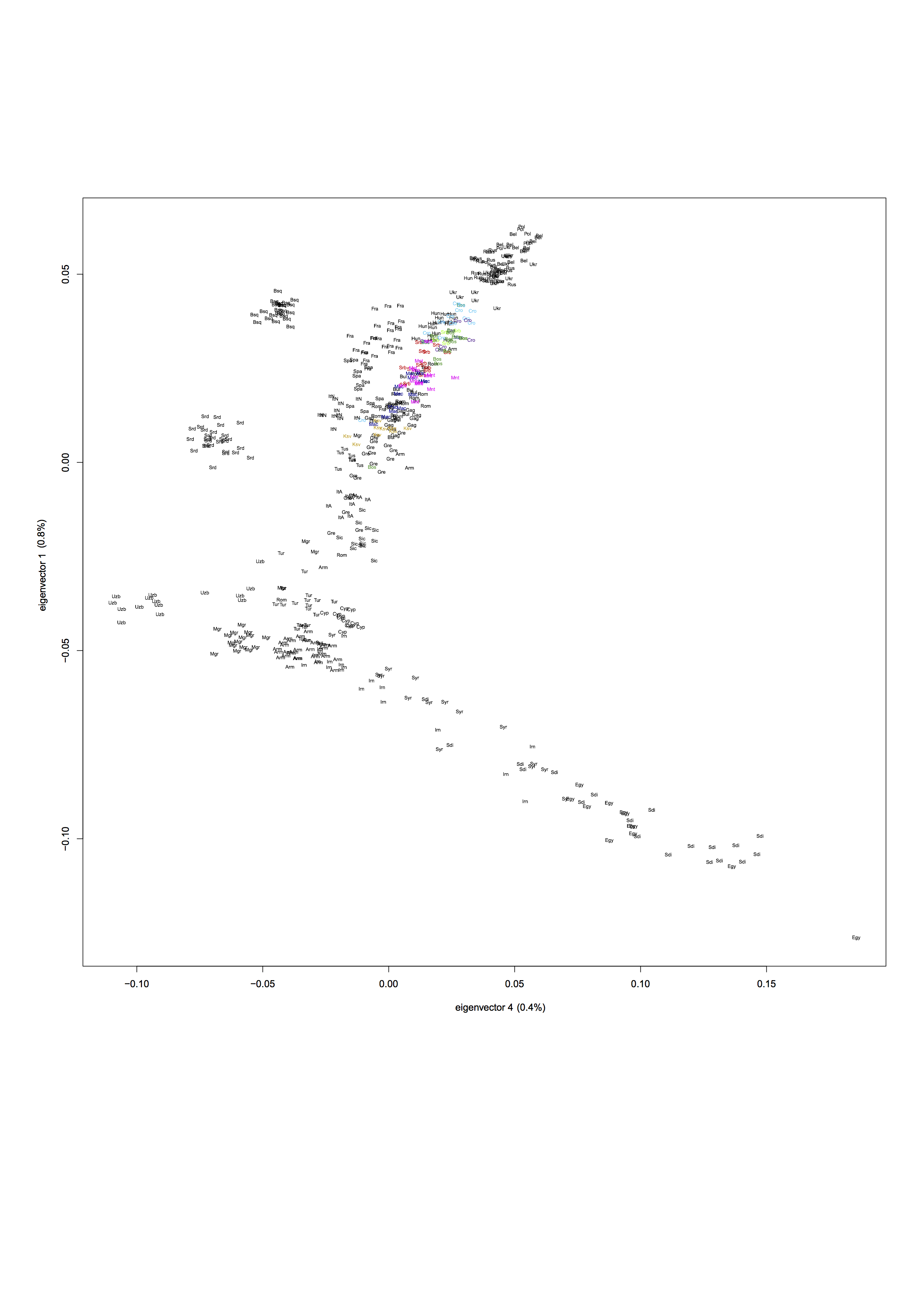

Supplement: Figure S4 — Principal component (PC) analysis (PC1 versus PC4) of the variation of autosomal SNPs in Western Balkan populations (highlighted) in Eurasian context (see Table S1 for population data and abbreviations). (TIFF) [file pone.0105090.s004.tiff]

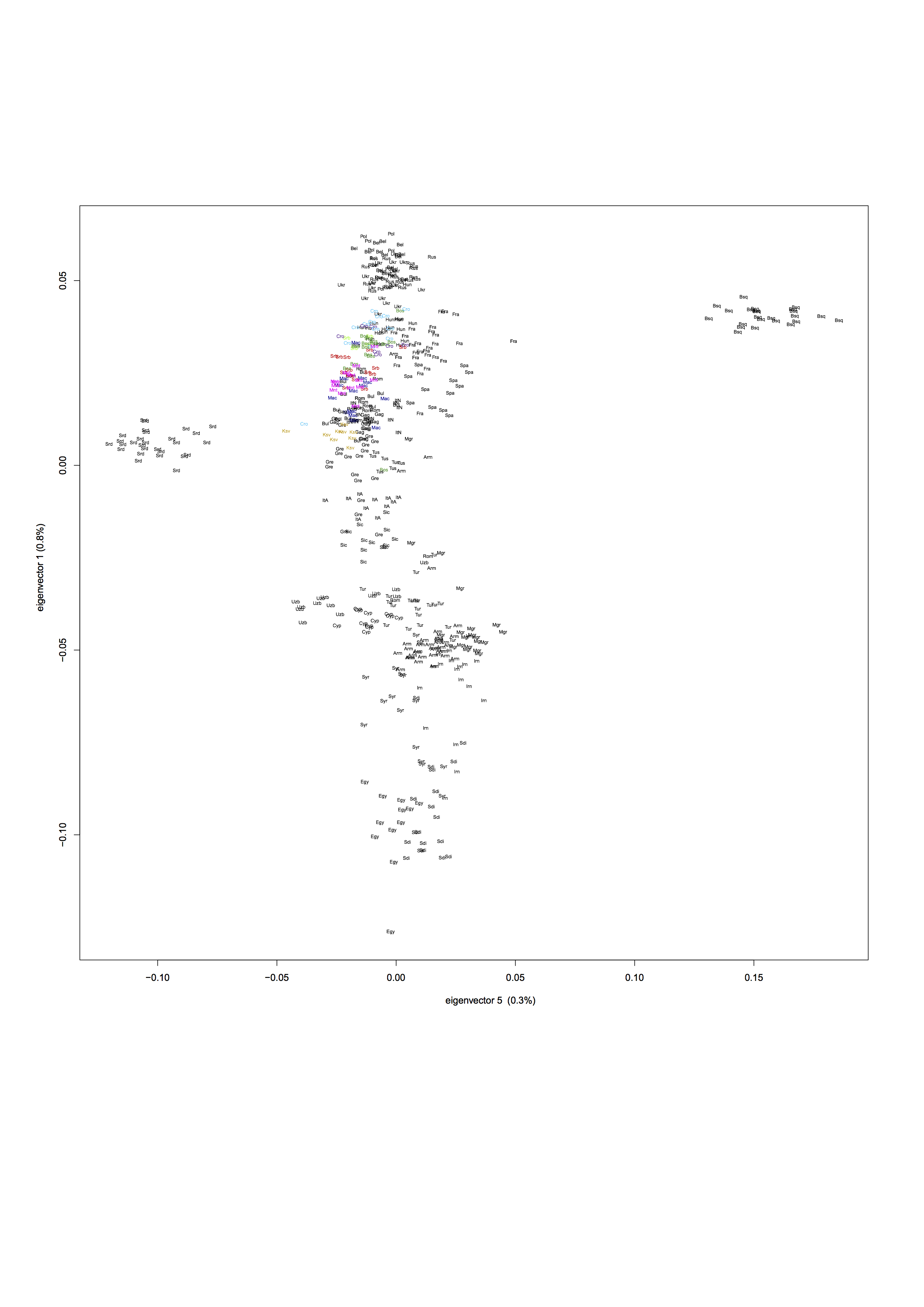

Supplement: Figure S5 — Principal component (PC) analysis (PC1 versus PC5) of the variation of autosomal SNPs in Western Balkan populations (highlighted) in Eurasian context (see Table S1 for population data and abbreviations). (TIF) [file pone.0105090.s005.tif]

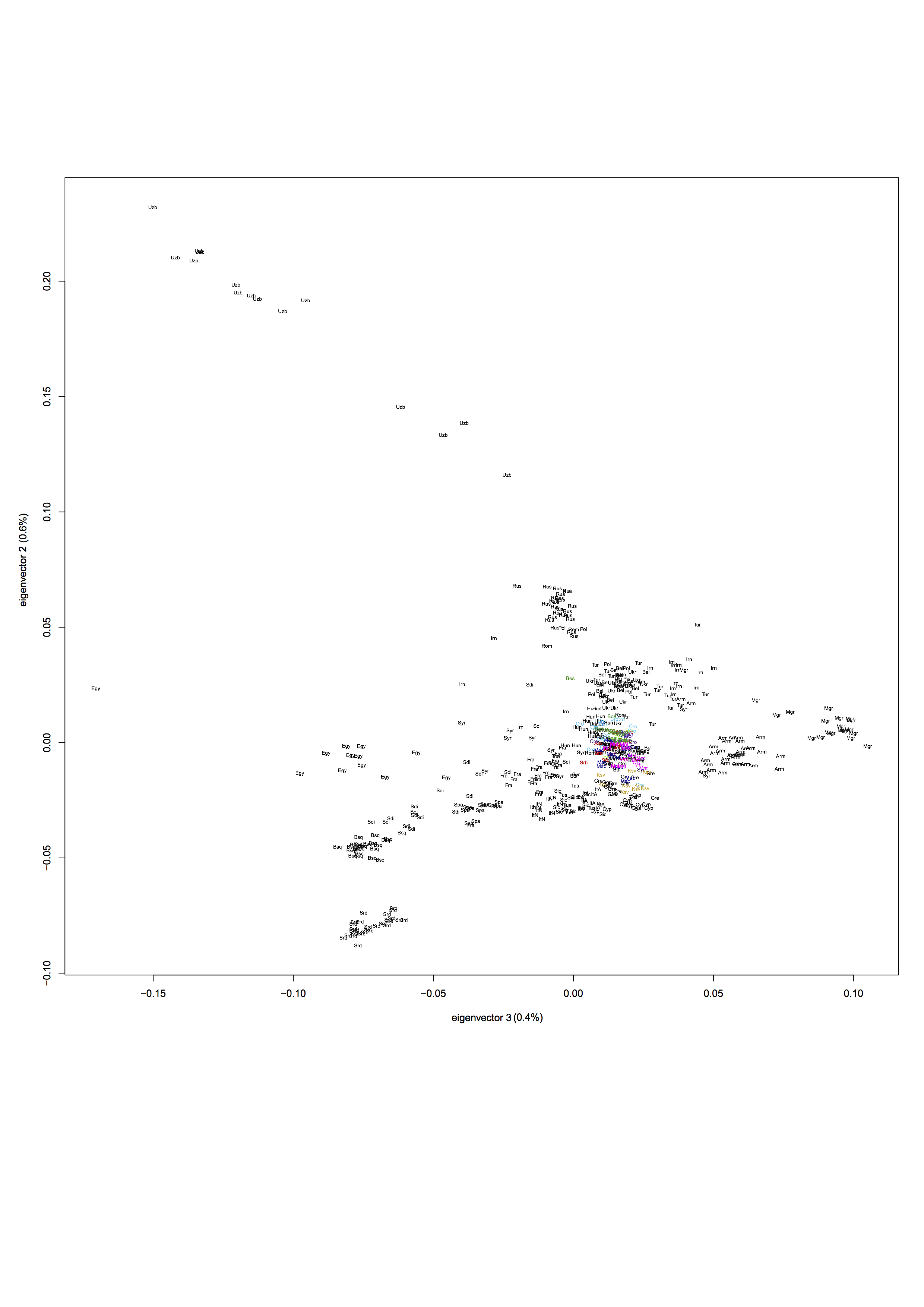

Supplement: Figure S6 — Principal component (PC) analysis (PC2 versus PC3) of the variation of autosomal SNPs in Western Balkan populations (highlighted) in Eurasian context (see Table S1 for population data and abbreviations). (TIF) [file pone.0105090.s006.tif]

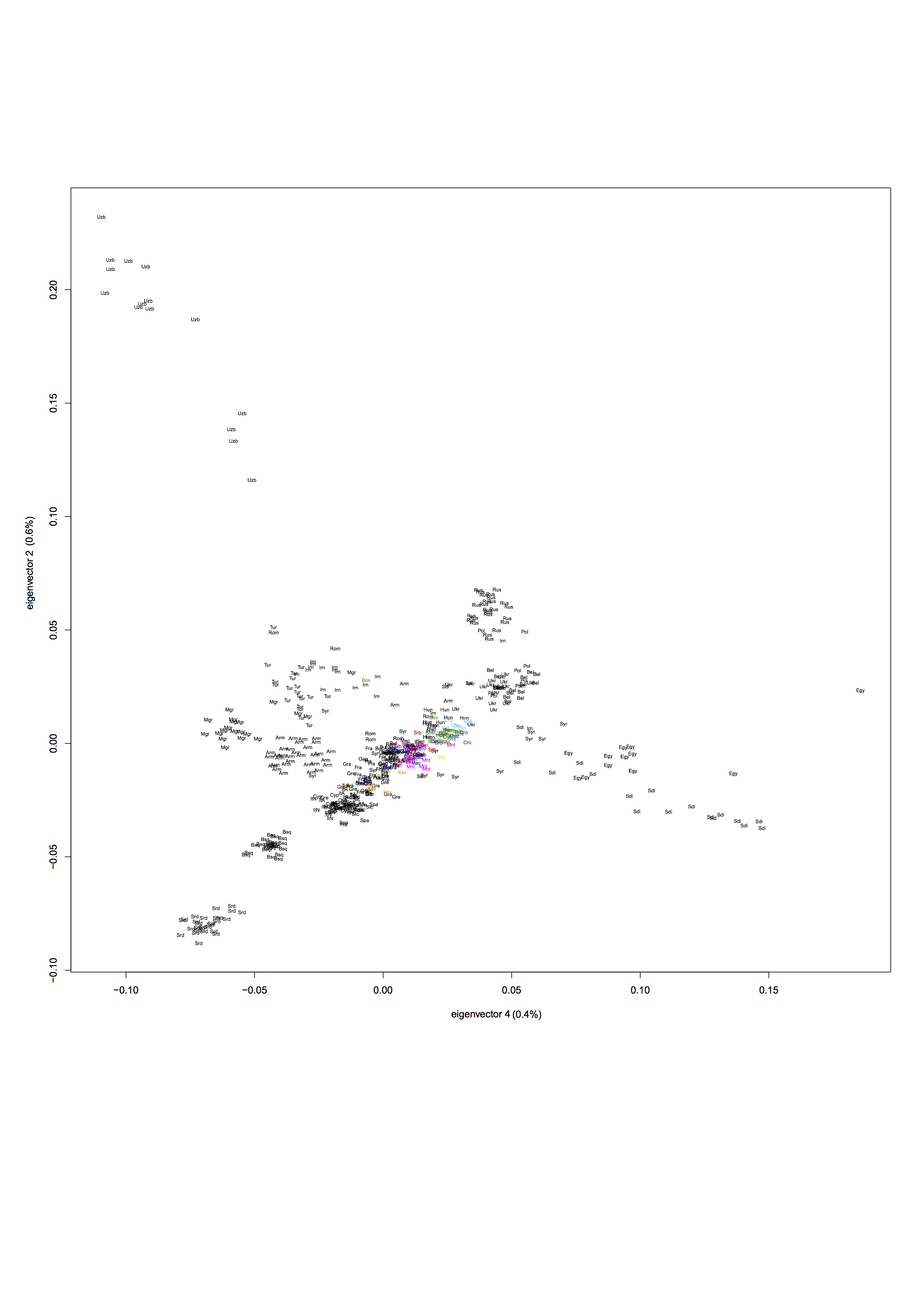

Supplement: Figure S7 — Principal component (PC) analysis (PC2 versus PC4) of the variation of autosomal SNPs in Western Balkan populations (highlighted) in Eurasian context (see Table S1 for population data and abbreviations). (TIF) [file pone.0105090.s007.tif]

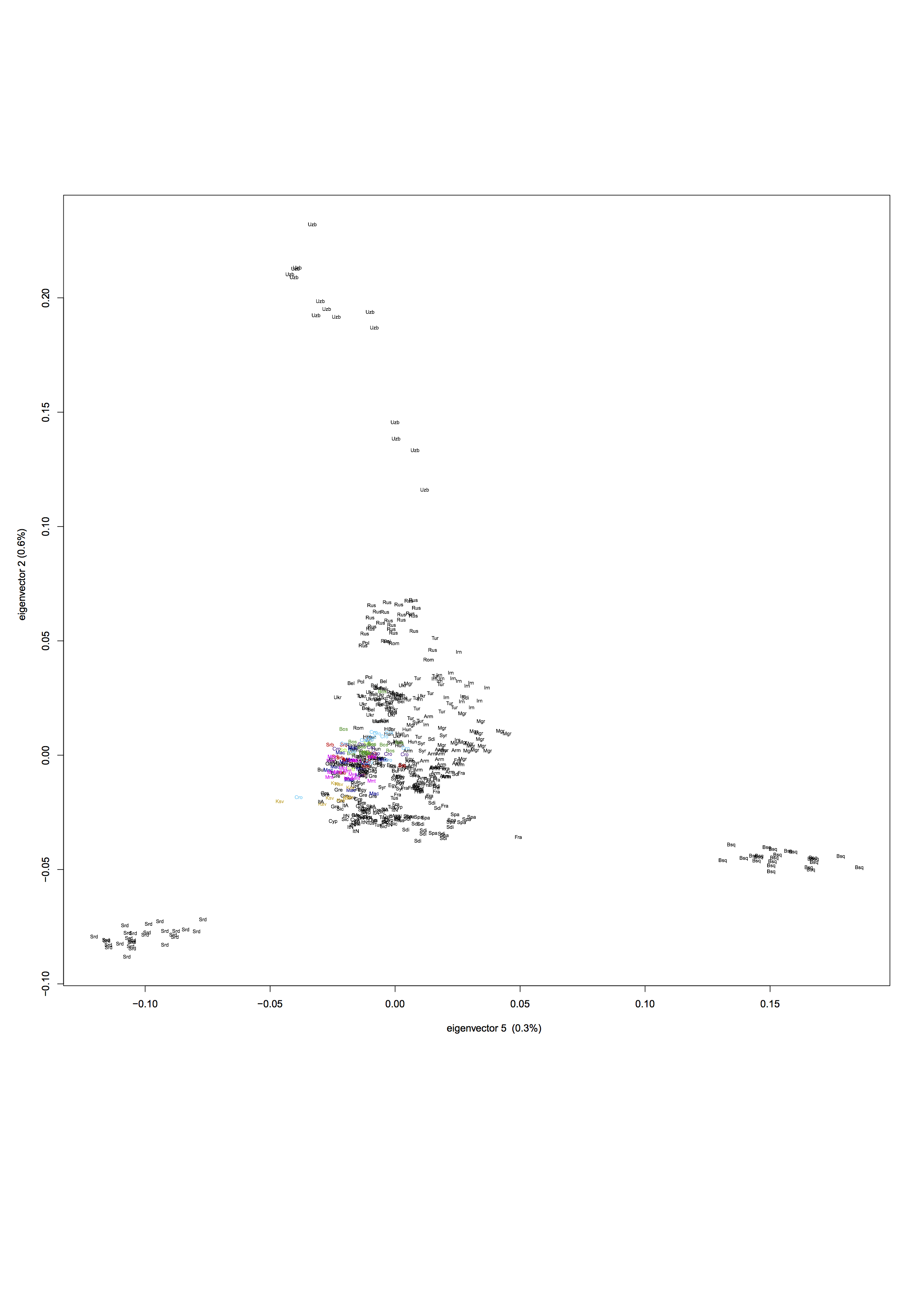

Supplement: Figure S8 — Principal component (PC) analysis (PC2 versus PC5) of the variation of autosomal SNPs in Western Balkan populations (highlighted) in Eurasian context (see Table S1 for population data and abbreviations). (TIF) [file pone.0105090.s008.tif]

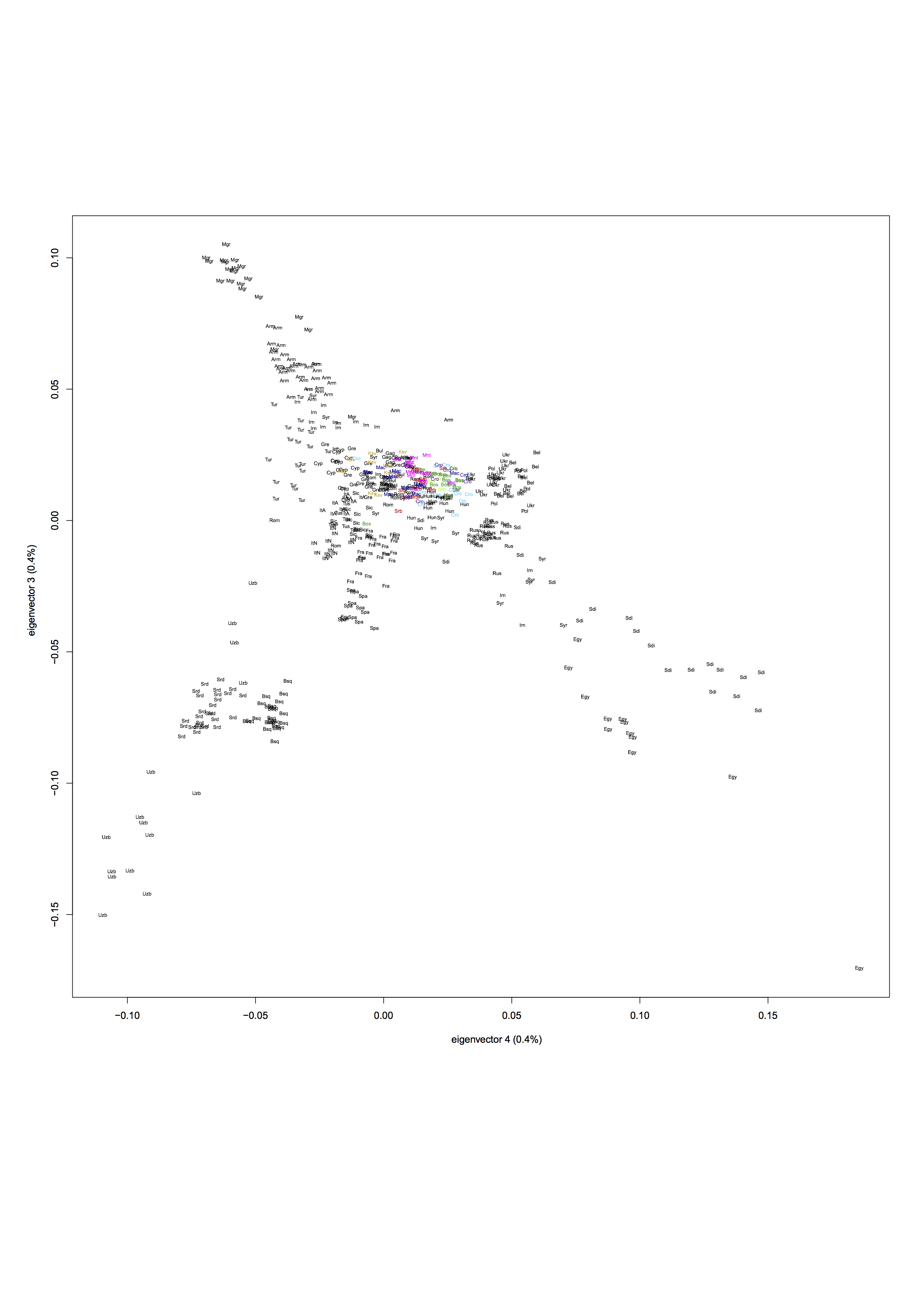

Supplement: Figure S9 — Principal component (PC) analysis (PC3 versus PC4) of the variation of autosomal SNPs in Western Balkan populations (highlighted) in Eurasian context (see Table S1 for population data and abbreviations). (TIF) [file pone.0105090.s009.tif]

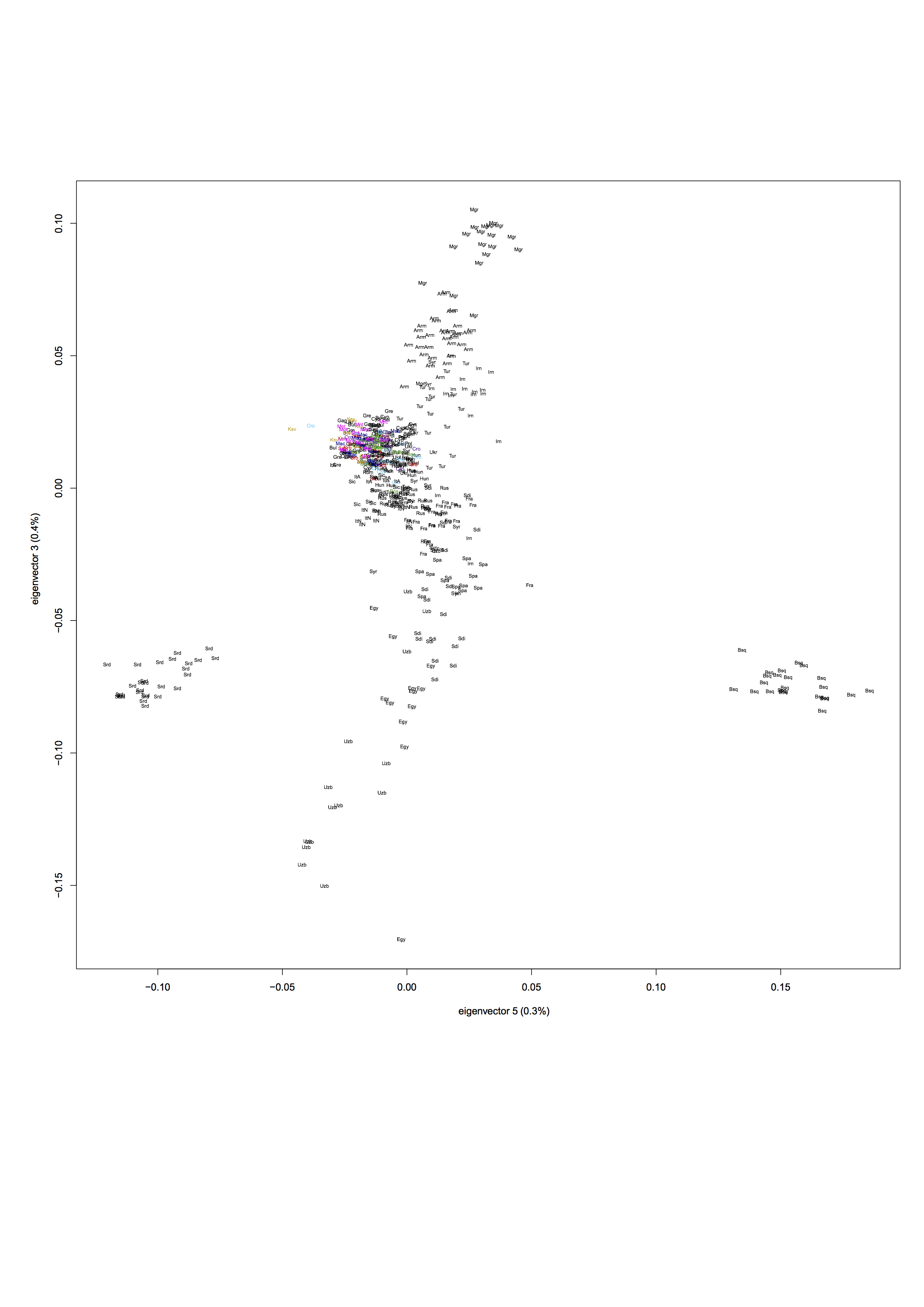

Supplement: Figure S10 — Principal component (PC) analysis (PC3 versus PC5) of the variation of autosomal SNPs in Western Balkan populations (highlighted) in Eurasian context (see Table S1 for population data and abbreviations). (TIF) [file pone.0105090.s010.tif]

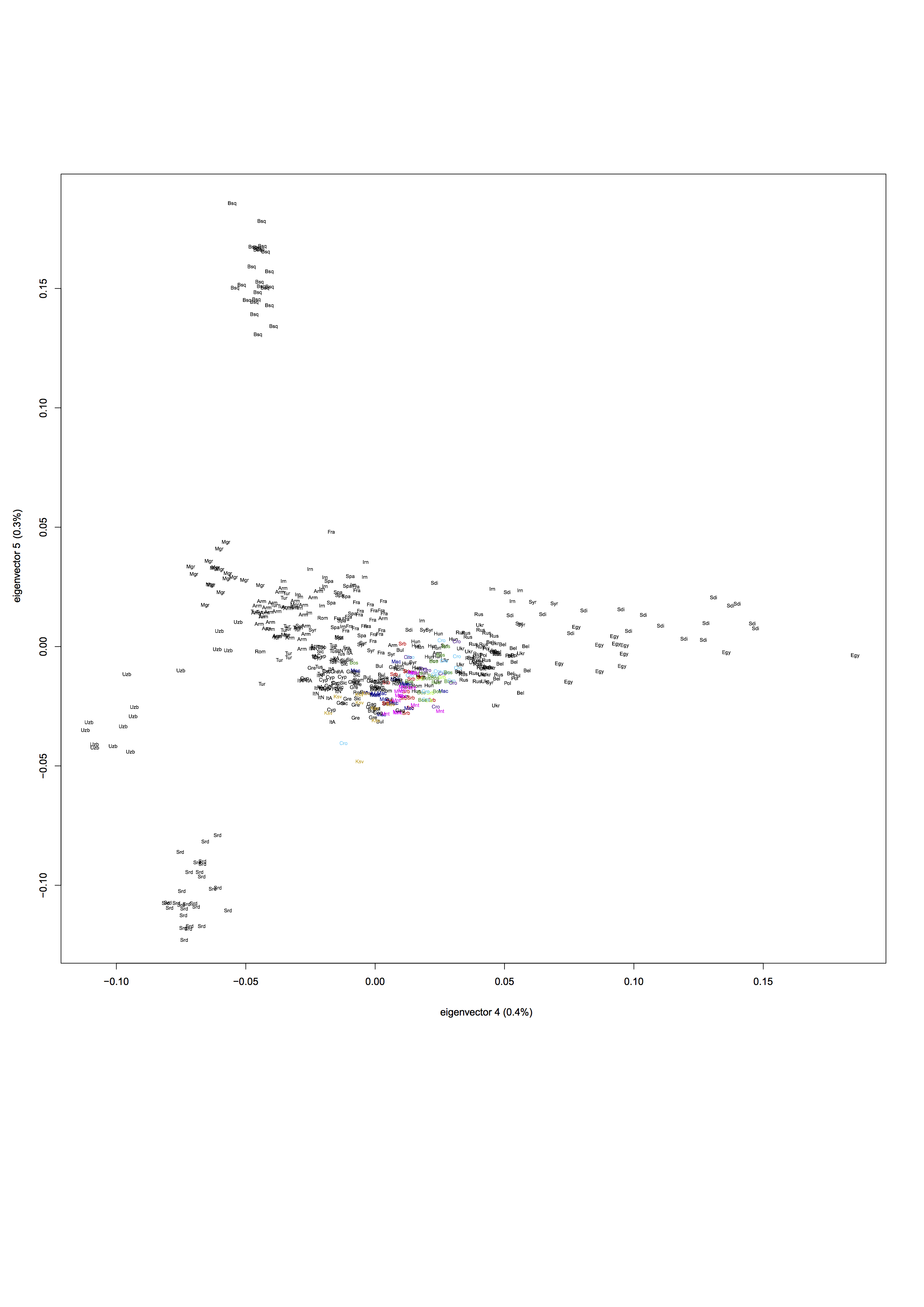

Supplement: Figure S11 — Principal component (PC) analysis (PC4 versus PC5) of the variation of autosomal SNPs in Western Balkan populations (highlighted) in Eurasian context (see Table S1 for population data and abbreviations). (TIF) [file pone.0105090.s011.tif]

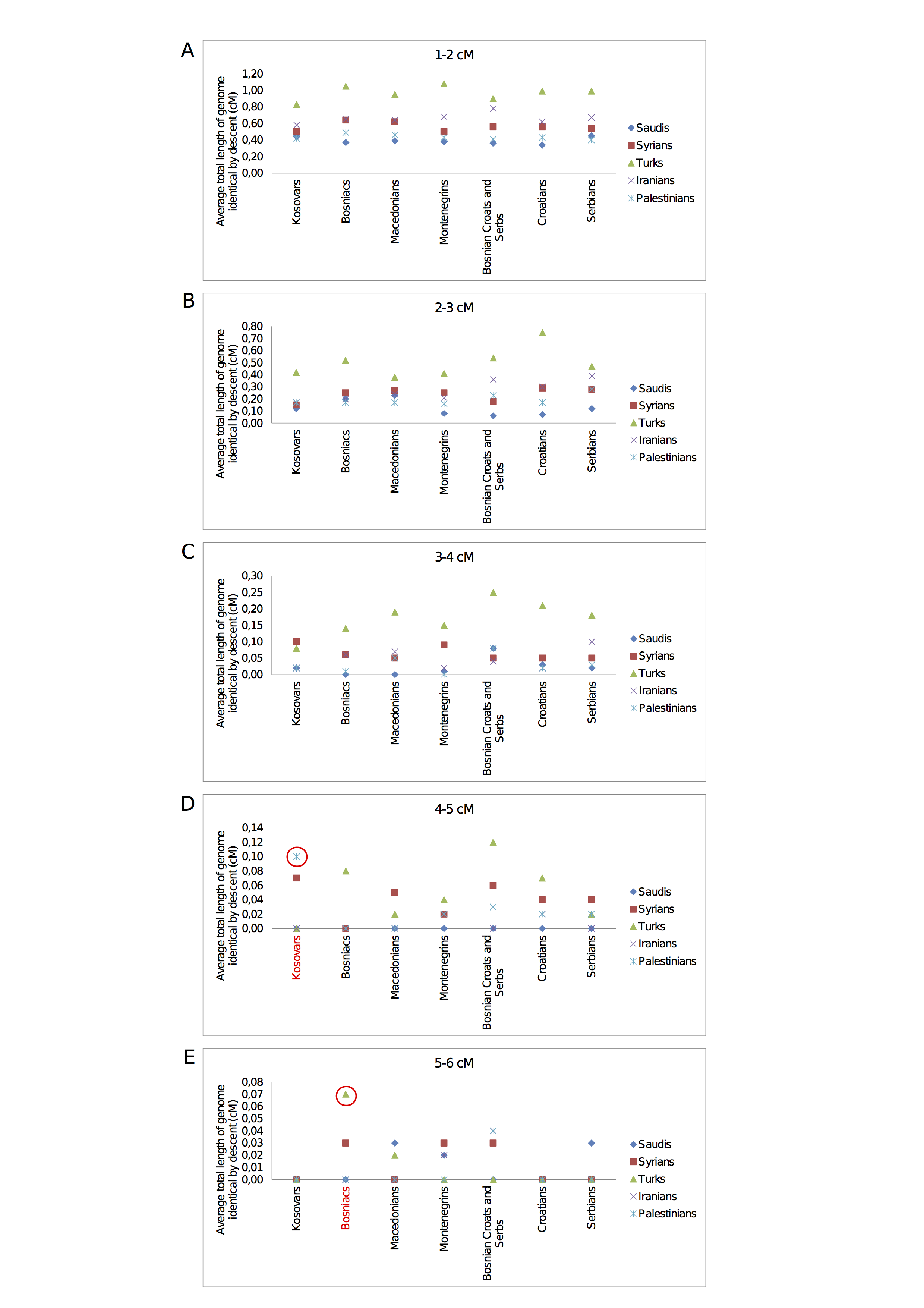

Supplement: Figure S12 — Principal component (PC) analysis based on the frequencies of mtDNA (panels A and C) and NRY haplogroups (panels B and D) of Western Balkan (WB) populations in a context of selected Central and South Europeans and Iranians from the Middle East (see the details of the dataset from Text S1). A: Pooled mtDNA data of WB populations. PC1 encompasses 36,6% and PC2 20,5% of total mtDNA variation; C: mtDNA data of each WB population plotted separately. PC1 encompasses 18,3% and PC2 16,3% of total mtDNA variation; B: Pooled NRY data of WB populations. PC1 encompasses 32,2% and PC2 24,4% of total NRY variation. D: NRY data of each WB population plotted separately. PC1 encompasses 28,3%, PC2 21,4% of total NRY variation. Abbreviations for studied WB populations are presented in Table S2, abbreviations for populations used for comparison are given in alphabetical order as follows: AUST - Austrians; BEL – Belarusians; BUL – Bulgarians; HUNG - Hungarians from Budapest; CZEC - Czechs; IR - Iranians; MAC.GRK - Macedonian Greeks; N. GRK - Greeks from North Greece; S.IT. - Italians from South Italy; N-E.IT - North-East Italians; ROM – Romanians; SLVK – Slovaks. Symbols on panels indicate geographical origin of populations as follows: triangles - Western Balkan; full circles - Central and East Europe; rhomboids - South Europe and Eastern Balkan; squares - Middle East. The references below are given in Text S1. The obtained NRY data were analyzed jointly with previously published data of 84 Bosniacs, 90 Bosnian Croats, 81 Bosnian Serbs, 118 Croatians, 64 Macedonian Albanians (FYROM Albanians pooled with Macedonians from FYROM) and 55 Albanians from Battaglia et al. 2009 [17], pooled with Kosovars, and 113 Serbians from Pericic et al. 2005 [12], pooled with Serbians and Montenegrins of this study. (TIF) [file pone.0105090.s012.tif]

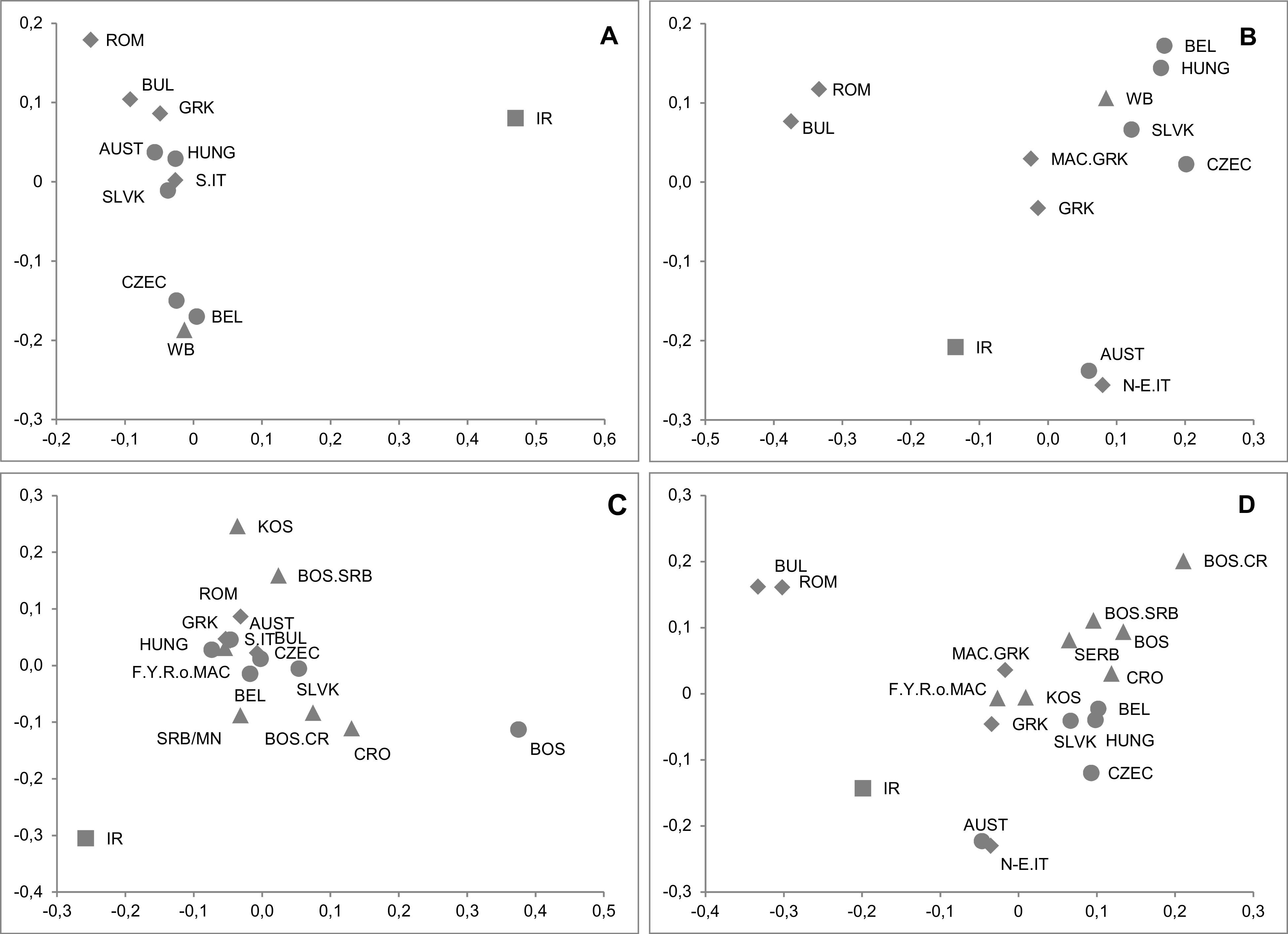

Supplement: Figure S13 — Average total length of genome shared identical by descent between Bosniacs, Kosovars and Near Eastern populations. Panels A-E indicate five length classes of ibd segments: 1–2, 2–3, 3–4, 4–5, 5–6 cM, respectively. Bosniacs and Kosovars are tested Muslim populations from Western Balkans; Macedonians, Montenegrins, Bosnian Croats and Serbs, Croatians, Serbians are non-Muslim populations from Western Balkans, used as a background. Red color of the Western Balkan population name and red circle around the symbol of Middle Eastern population indicates significantly higher ibd sharing between these populations as compared to non-Muslim background. (TIF) [file pone.0105090.s013.tif]

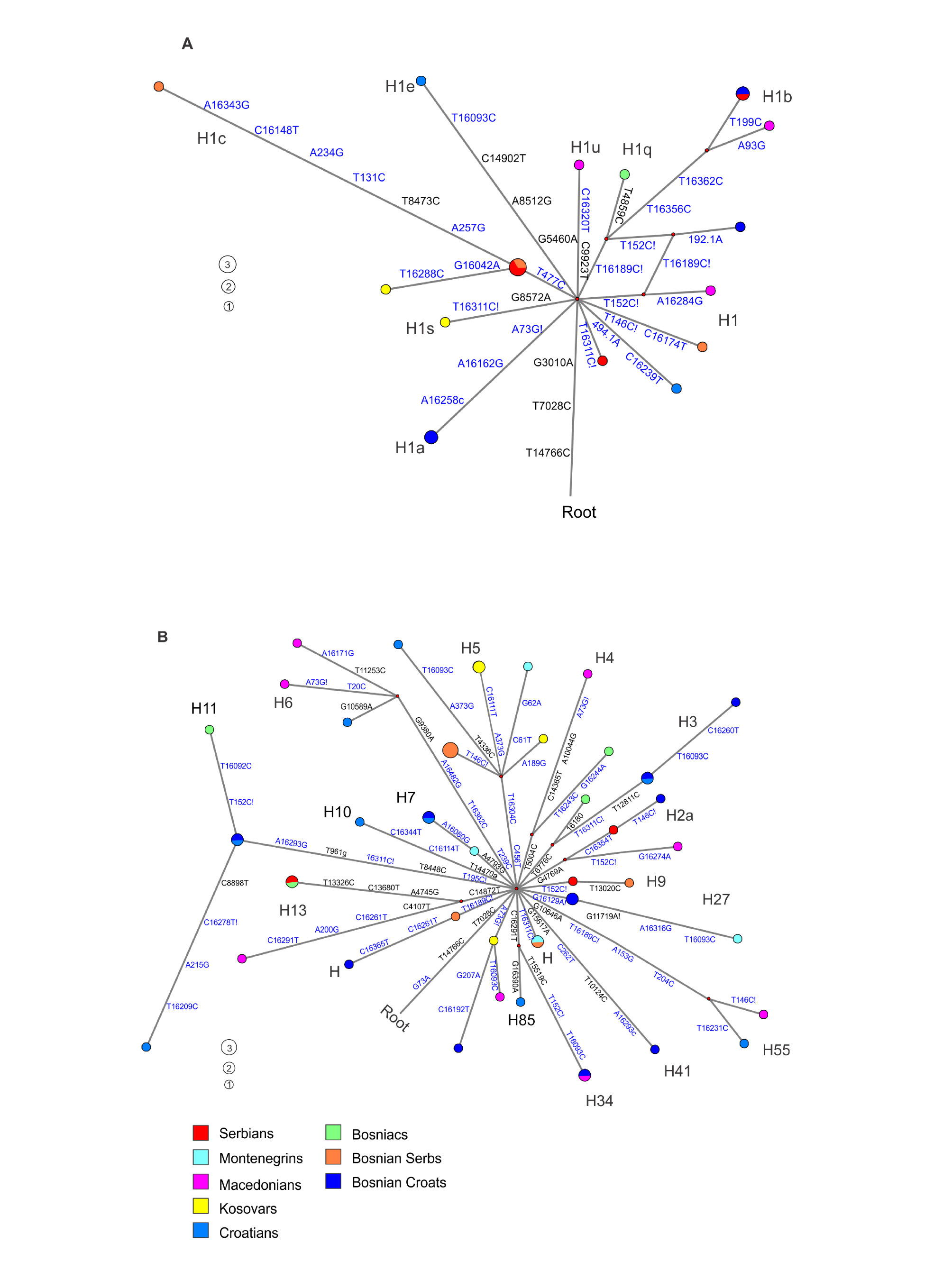

Supplement: Figure S14 — Median-joining network of mtDNA hg H lineages of Western Balkan populations: A: subhg H1 and its sub-branches; B: other subhgs of hg H. A total number of 19 H1 and 49 other H haplotypes are reported. Numbers on links indicate the mutations: blue color indicates HVS1 and HVS2 mutations, black color coding region mutations. Polymorphic nucleotide sites are numbered according to Reconstructed Sapiens Reference Sequence. Node size is proportional to absolute haplotype frequency, as it is reported in figure legend. (TIFF) [file pone.0105090.s014.tif]

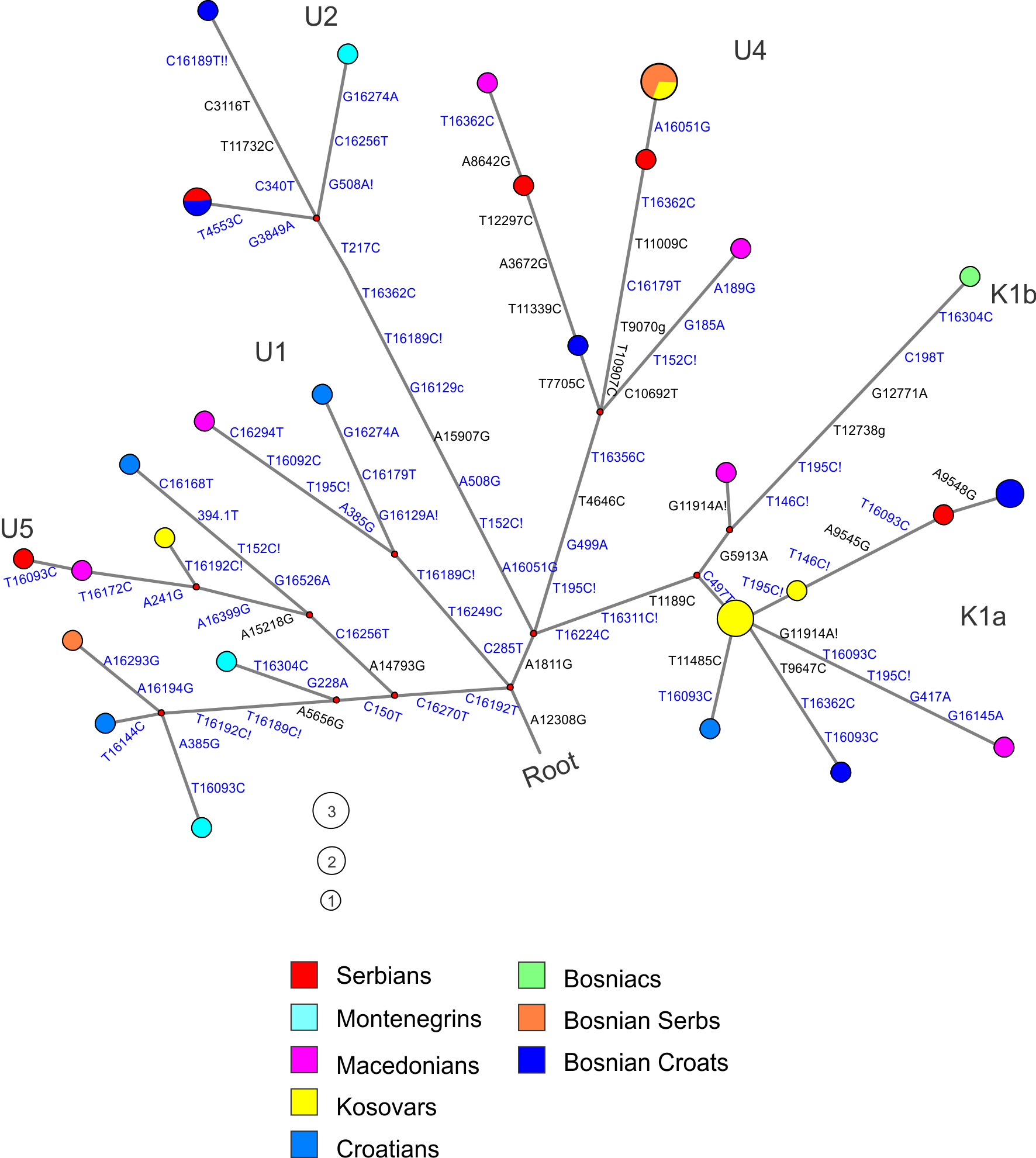

Supplement: Figure S15 — Median joining network of mtDNA hg U lineages. A total number of 34 U haplotypes are presented. A diagnostic mutation A1811G has been added into the network, but not genotyped in the sample. For further details, see the legend of figure S5. (TIF) [file pone.0105090.s015.tiff]

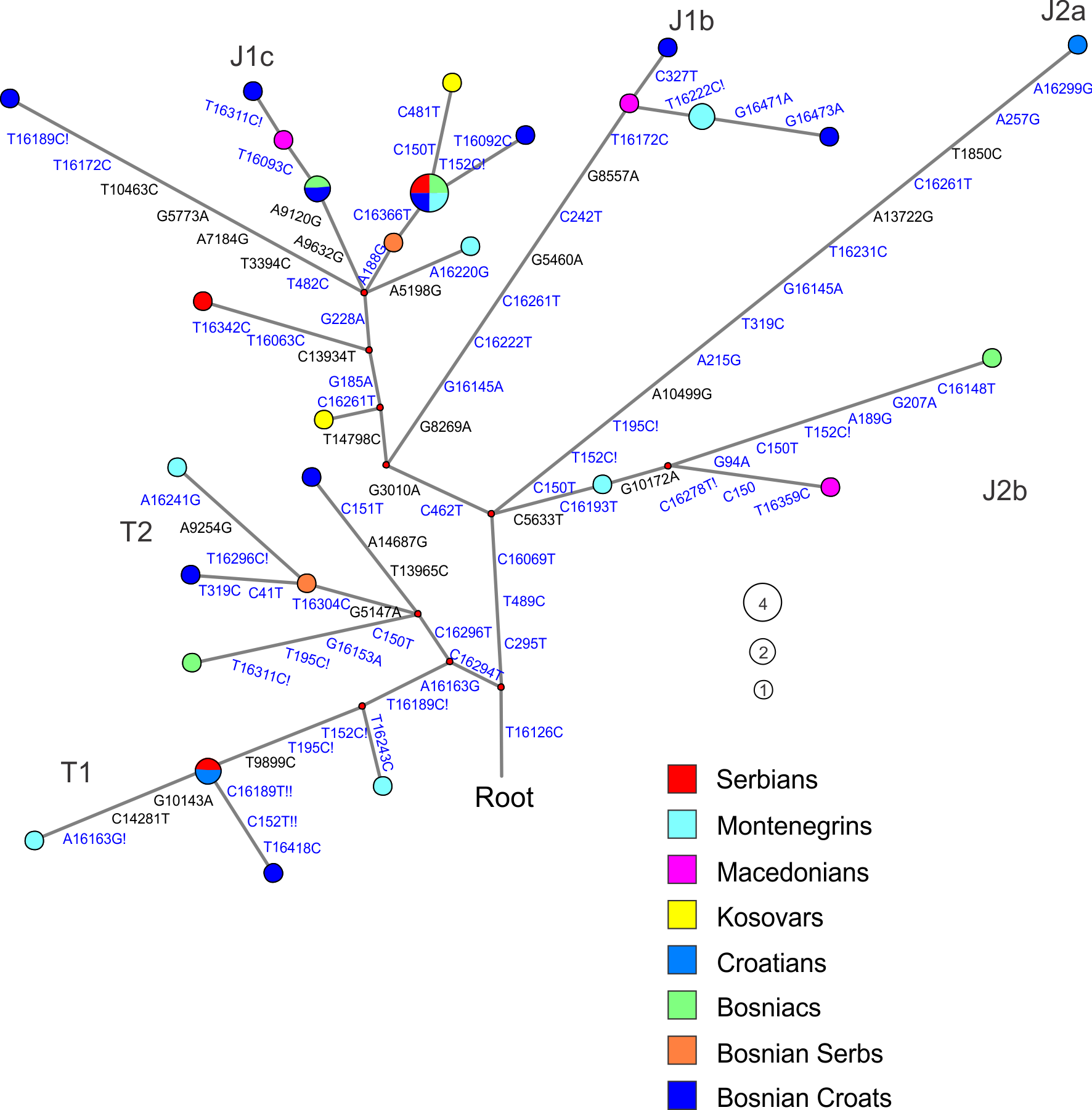

Supplement: Figure S16 — Median joining network of mtDNA lineages for hgs J and T in the Western Balkan region. A total number of 24 J and 10 T haplotypes are presented. For further details, see the legend of figure S5. (TIF) [file pone.0105090.s016.tif]

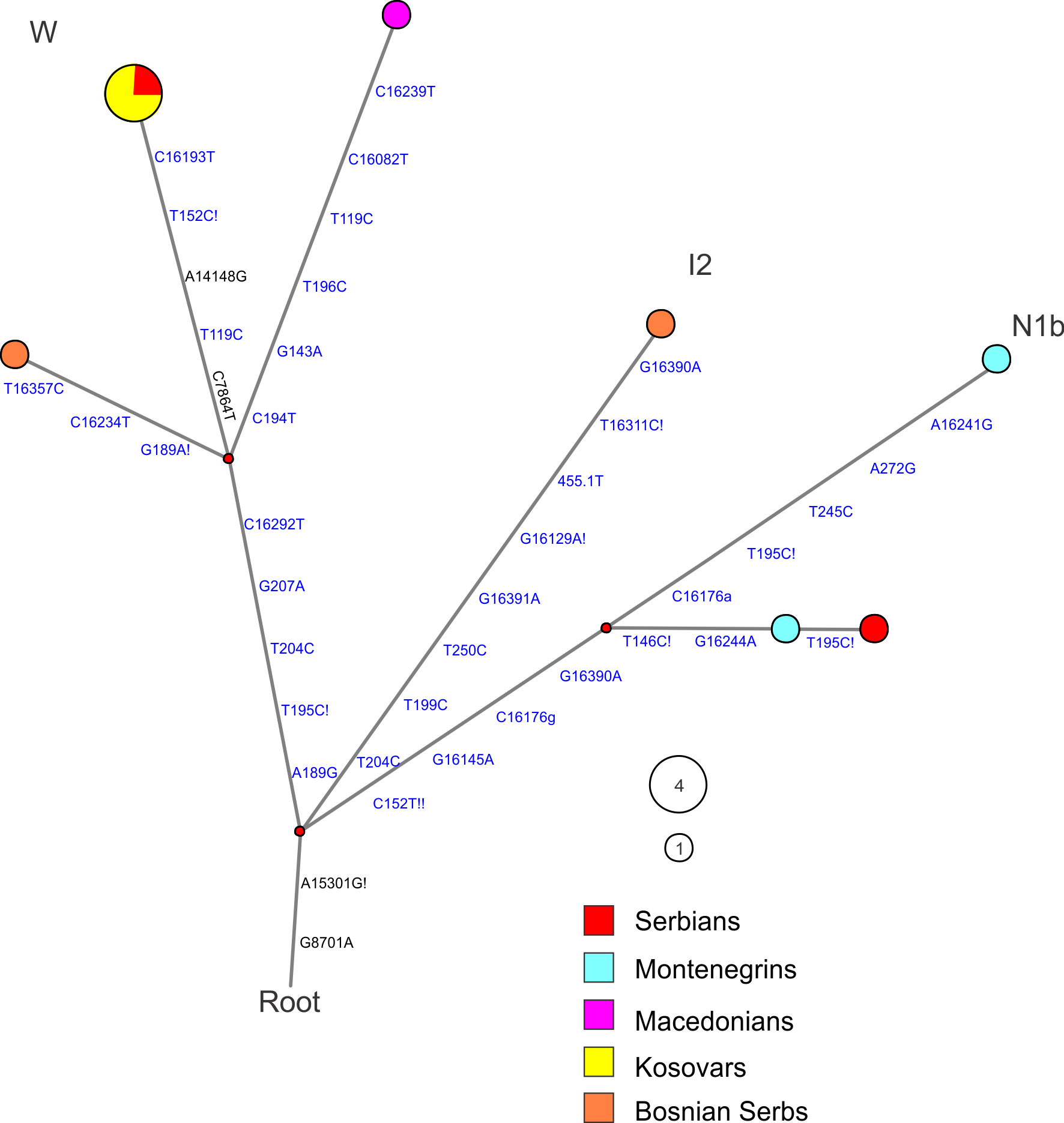

Supplement: Figure S17 — Median joining network of mtDNA lineages of hgs HV, V and R0a. A total number of 11 V, 8 HV and 2 R0a haplotypes are reported.For further details, see the legend of figure S5. (TIF) [file pone.0105090.s017.tif]

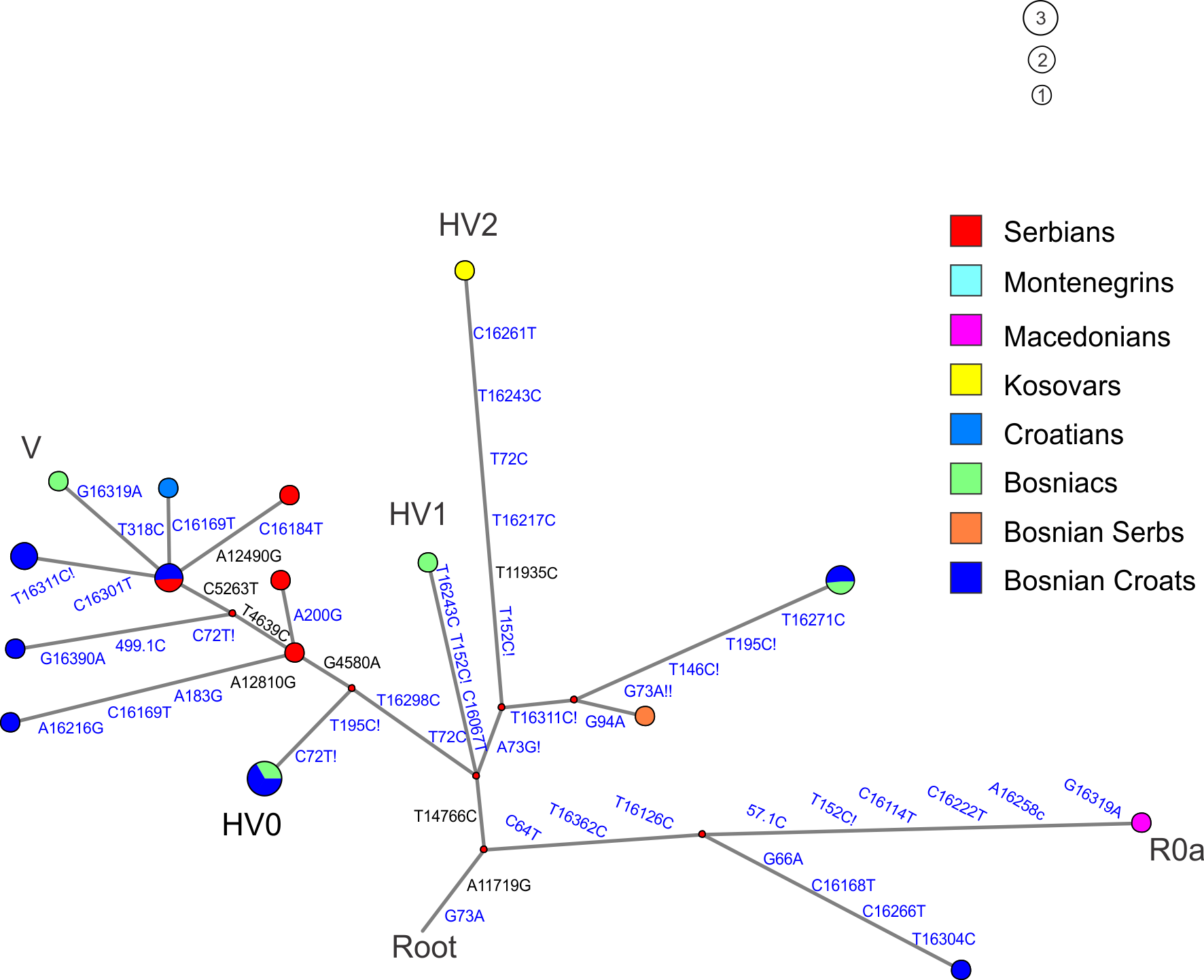

Supplement: Figure S18 — Median joining network of mtDNA lineages from hgs W, I and N1b. A total number of 6 W, 1 I and 3 N1b of haplotypes are reported. For further details, see the legend of figure S5. (TIF) [file pone.0105090.s018.tif]

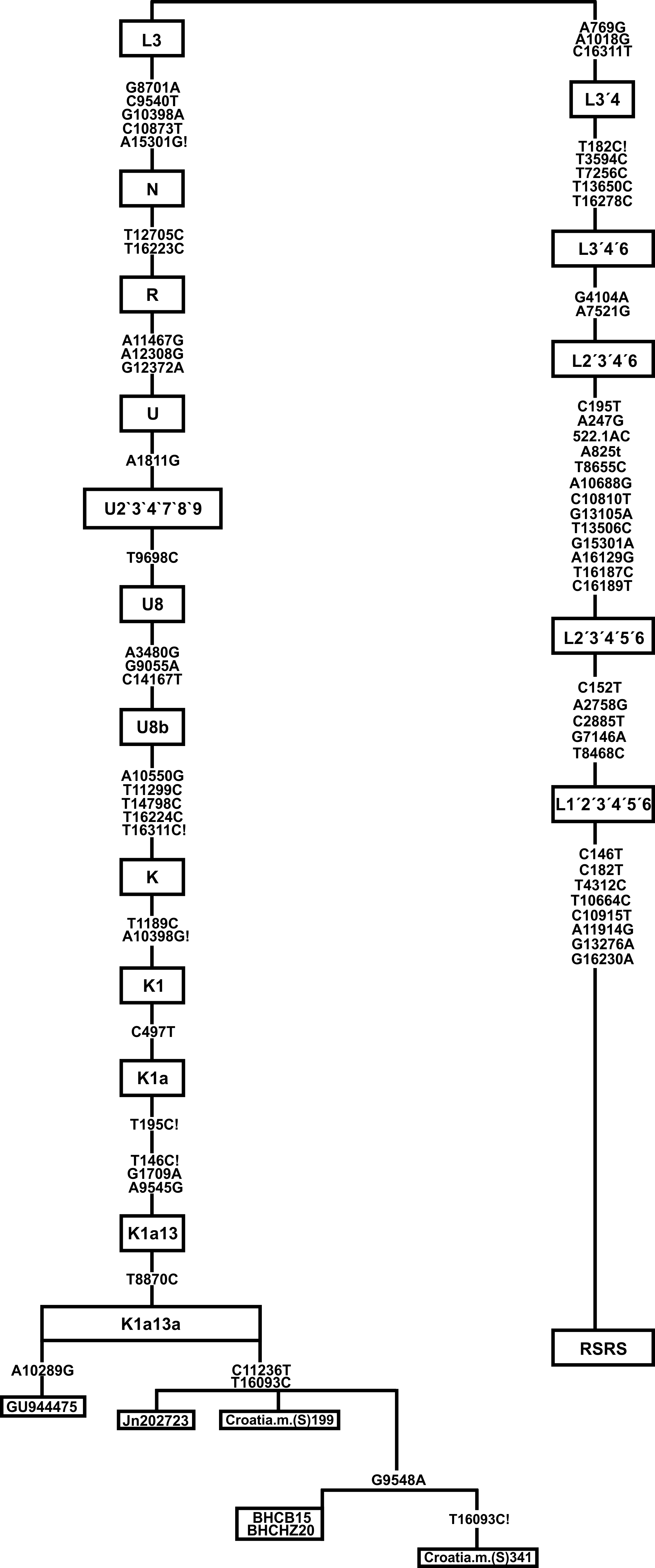

Supplement: Figure S19 — Phylogenetic tree of mtDNA K1a13a complete sequences. Two samples of Bosnian Croats (BHCB15 and BHCHZ20), and two from Croatia [Croatia.m.(S)199 and Croatia.m.(S)34] are sequenced in this study, the others are from Phylotree mtDNA Build 15. The mutations are given relative to the Reconstructed Sapiens Reference Sequence. (TIF) [file pone.0105090.s019.tif]

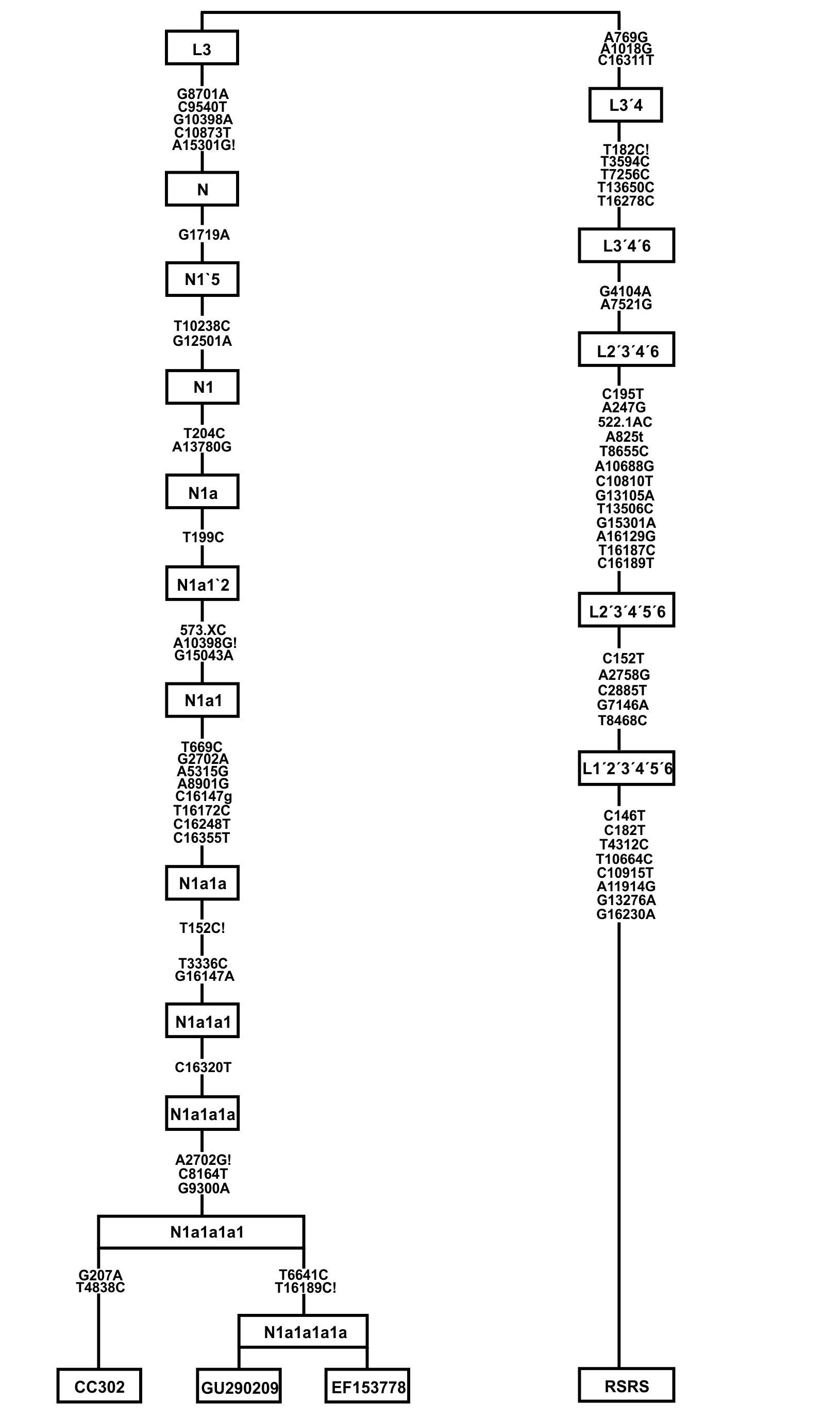

Supplement: Figure S20 — Phylogenetic tree of mtDNA N1a complete sequences. One sequence of Croatian from Croatia is sequenced in this study, the others are from Phylotree mtDNA Build 15. The mutations are given relative to the Reconstructed Sapiens Reference Sequence. (TIF) [file pone.0105090.s020.tif]

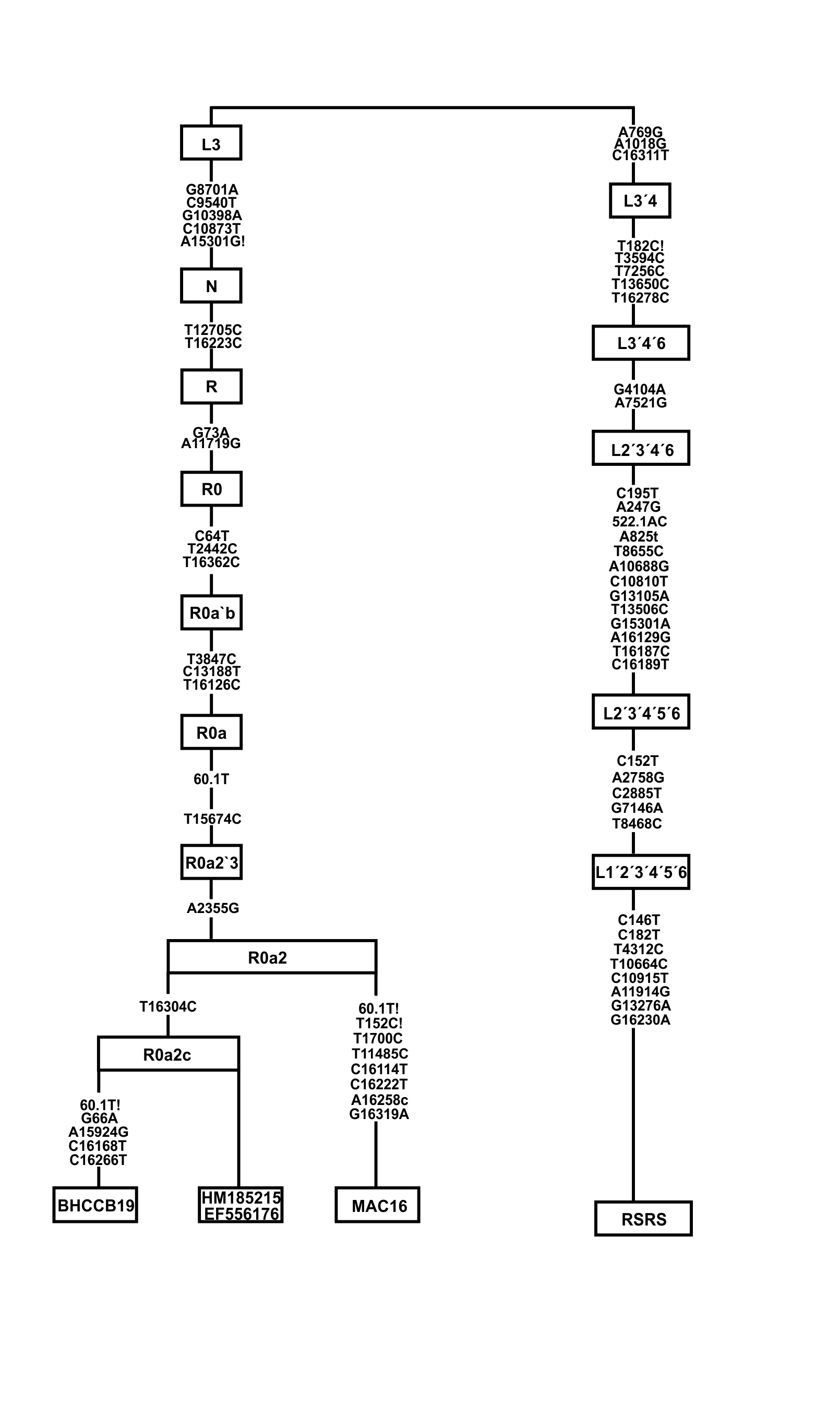

Supplement: Figure S21 — Phylogenetic tree of mtDNA R0a2 complete sequences. Macedonian (MAC16) and Croat from Bosnia and Herzegovina (BHCCB19) are sequenced in this study, the others are from Phylotree mtDNA Build 15. The mutations are given relative to the Reconstructed Sapiens Reference Sequence. (TIF) [file pone.0105090.s021.tif]
