# Supplementary material for: Standing at the Gateway to Europe - The Genetic Structure of Western Balkan Populations Based on Autosomal and Haploid Markers
Source: PLoS One. 2014 Aug 22;9(8):e105090. doi: 10.1371/journal.pone.0105090 (PMC4141785; doi:10.1371/journal.pone.0105090)
Supplement: Text S1 — Description of the sample and methods of the analyses of mtDNA and NRY. (DOCX) [file pone.0105090.s032.docx]

**Supplementary Text 1. Description of the sample and methods of the analyses of mtDNA and NRY.**

**The sample for mtDNA and NRY analyses**

Blood samples were collected from 175 unrelated and healthy adult individuals of both sexes, residing in Bosnia and Herzegovina (n=77), Croatia (mainland, Zagreb region, n=19), Serbia (Belgrade area, n=21), Monte Negro (Podgorica, n=18), Kosovo (Pristina and Prizren, n=18) and Macedonians from the F.Y. Republic of Macedonia (Skopje, n=22) (Figure 1). The sample of 77 individuals from Bosnia and Herzegovina consisted of 17 Bosniacs (Sarajevo and Zavidovici), 41 Bosnian Croats (20 from Central Bosnia - Zepce and Maglaj - and 21 from South Bosnia and Herzegovina - Mostar, Grude, Livno and Capljina) and 19 Bosnian Serbs (Doboj and Banjaluka region).Out of the 175 Western Balkan samples, all were genotyped for mtDNA variation and 85 were genotyped for NRY variation. 7 mtDNA samples of our sample set were sequenced completely (Figures S19-21), completely sequenced mitochondrial genomes are available in the National Center for Biotechnology Information (NCBI), in Genbank (accession numbers KM103653 - KM103659). 70 individuals were randomly chosen for autosomal genotyping of 660 000 genome-wide markers in this study, 20 Croatian samples of this sample set has been characterized for autosomal variation in Behar et al. [1].

We have used the published data of 3202 samples of mtDNA from the European and Middle Eastern populations to put the mtDNA variation of 175 Western Balkan samples of this study (Table S5) into a wider context. The total mtDNA sample used for principal component analysis (PCA, Figure S13A and C) consisted of 273 Austrians [2], 267 Belarusians [3], 179 Czechs [4], 374 Slovaks [5], 211 Hungarians [6], 319 North Greeks [7], 341 South Italians [8], 94 Romanians [9], 996 Bulgarians [9]and 148 Iranians [10].

The Western Balkan NRY sample consisted of 9 Bosniacs, 14 Bosnian Serbs, 29 Bosnian Croats (8 from Central Bosnia, 21 from Herzegovina), 3 Croatians, 11 Serbians, 7 Montenegrins, 6 Macedonians of the Republic of Macedonia and 6 Kosovars. Due to the small individual sample sizes, the obtained NRY data were analyzed jointly with previously published NRY data of 84 Bosniacs, 90 Bosnian Croats, 81 Bosnian Serbs, 118 Croatians, 64 Macedonian Albanians (FYROM Albanians) and 55 Albanians from Battaglia et al. [11], and 113 Serbians from Pericic [12] for PCA (Table S6). The NRY sample of a wider context consisted of 2424 samples of 258 Austrians[13],565 Belarusians [3], 149 Greeks (57 from the former Yugoslav Republic of Macedonia, and 92 from Athens, Greece), 67 northeast Italians, 75 Czechs and 53 Hungarians from Battaglia et al.[11], 150 Slovaks [14], 149 Romanians and 808 Bulgarians from Karachanak et al. [15]and 150 Iranians from Regueiro et al. [16] (Figure S13B, D).

**Materials and methods**

***MtDNA and NRY genotyping***

Observed maternal lineages were determined into the hgs by the sequencing of the first and the second hypervariable segment (HVS1 and HVS2) of mtDNA control region and by the use of the high-resolution genotyping. The sequences of mtDNA HVS1 and HVS2 regions between nucleotide positions (nps) 16020 and 16519 and 29 and 510, respectively, as well as relevant coding region sequences of mtDNA and sequences of NRY were amplified by Biometra T1 Thermocycler 96 (Biometra GmbH, Goettingen, Germany)  and sequenced by ABIPrism 3130xl Genetic Analyser (Applied Biosystem, Foster City, CA, USA). The mtDNA samples were first analyzed for diagnostic nps of control region and thereafter hierarchically genotyped for putative hg-specific coding region mutations by RFLP or by direct sequencing, following the most recent established classification and nomenclature available at [www.phylotree.org](http://www.phylotree.org)[17]. The protocols of amplification and sequencing of mtDNA HVS1, HVS2 and coding region sequences were performed as described in [18–21]. All sequences were aligned and analysed by the use of ChromasPro software (Technelysium Pty Ltd). The complete sequencing of mtDNA genomes was done according to the procedures and conditions described in Rieder et al. [22]. The frequencies of mtDNA haplogroups and the list of the mtDNA HVS1 and HVS2 haplotypes observed in the sample of three ethnic groups of Bosnia and Herzegovina and in other studied Western Balkan populations are presented in Table S5.

The phylogenetic analysis of mtDNA haplotypes (Figures S14-18) was carried out by the use of the software Network 4.5.0.2 and Network Publisher (<http://www.fluxus-engineering.com>). The principle of maximum parsimony was applied using a reduced median algorithm (r = 2) [23], followed by a median joining algorithm (ε = 0) [24] and corrected by hand, if needed. The weights of mutations were assigned according to their observed rate of evolution [25–27].

The NRY variation (Table S6) was genotyped with the set of biallelic markers either by determining the restriction fragment length polymorphisms (RFLP) or sequencing, according to the current NRY phylogeny by Battaglia et al.[28] and Karafet et al. [29].

PCA based on the frequencies of mtDNA and NRY hgs was performed by the use of the software POPSTR (http://harpending.humanevo.utah.edu/popstr/). The results are given in Figure S13.

***Statistical analysis***

MtDNA HVS1 sequences were used to calculate the number of haplotypes and polymorphic sites, haplotype diversity, mean number of pairwise differences and nucleotide diversity of the populations (AMOVA) by the use of software Arlequin, v3.5 for all studied populations (Table S7). For testing the genetic structure of the Western Balkan populations, AMOVA was performed by the use of mtDNA HVS1 haplotypes (Tables S5 and S7).Genetic distances were estimated using the haplogroup (for Mantel test) or HVS-I haplotype frequency (for AMOVA) based linearized F_ST_-s [30]. Mantel test with 10000 permutation steps was used to test the correlation between the genetic and geographic distances. In order to generate geographical matrix, we used program Geographic Distance Matrix Generator v1.2.3. [31]*.* For AMOVA (Table S9) and Mantel test (Table 1) the populations were grouped first according to their geographic and thereafter to their linguistic and religious affiliations – the last grouping was done as follows: Kosovars were grouped with Bosniacs to the Islamic (Muslim); Croats, both from Croatia and from Bosnia and Herzegovina into Catholic; Macedonians of former Yugoslav Republic of Macedonia, Montenegrins, Bosnian Serbs and Serbians into Orthodox group.

Bayesian 95% credible regions (CRs) for haplogroup frequencies were calculated with the computer program SAMPLING, provided by Dr. Vincent Macaulay.The coalescence time estimates and their standard deviations of mtDNA haplotypes were calculated according to Forster et al. [32] and Saillard et al. [33]. Estimated coalescense ages for the largest hgs found in studied Western Balkan populations are presented in Table S10.

**References**

1. Behar D, Metspalu M, Baran Y, Kopelman N, Yunusbayev B, et al. (2014) No Evidence from Genome-Wide Data of a Khazar Origin for the Ashkenazi Jews. Human Biology (in press).

2. Brandstätter A, Niederstätter H, Pavlic M, Grubwieser P, Parson W (2007) Generating population data for the EMPOP database - an overview of the mtDNA sequencing and data evaluation processes considering 273 Austrian control region sequences as example. Forensic Sci Int 166: 164–175. Available: http://www.ncbi.nlm.nih.gov/pubmed/16829006. Accessed 2 November 2012.

3. Kushniarevich A, Sivitskaya L, Danilenko N, Novogrodskii T, Tsybovsky I, et al. (2013) Uniparental genetic heritage of belarusians: encounter of rare middle eastern matrilineages with a central European mitochondrial DNA pool. PloS one 8: e66499. Available: http://www.pubmedcentral.nih.gov/articlerender.fcgi?artid=3681942&tool=pmcentrez&rendertype=abstract. Accessed 25 June 2013.

4. Malyarchuk BA, Vanecek T, Perkova MA, Derenko M V, Sip M (2006) Mitochondrial DNA variability in the Czech population, with application to the ethnic history of Slavs. Hum Biol 78: 681–696. Available: http://www.ncbi.nlm.nih.gov/pubmed/17564247. Accessed 28 November 2012.

5. Lehocký I, Baldovic M, Kádasi L, Metspalu E (2008) A database of mitochondrial DNA hypervariable regions I and II sequences of individuals from Slovakia. Forensic Sci Int-Gen 2: e53–9. Available: http://www.ncbi.nlm.nih.gov/pubmed/19083829. Accessed 28 November 2012.

6. Irwin J, Egyed B, Saunier J, Szamosi G, O’Callaghan J, et al. (2007) Hungarian mtDNA population databases from Budapest and the Baranya county Roma. Int J Legal Med 121: 377–383. Available: http://www.ncbi.nlm.nih.gov/pubmed/17186294. Accessed 2 November 2012.

7. Irwin J, Saunier J, Strouss K, Paintner C, Diegoli T, et al. (2008) Mitochondrial control region sequences from northern Greece and Greek Cypriots. Int J Legal Med 122: 87–89. Available: http://www.ncbi.nlm.nih.gov/pubmed/17492459. Accessed 2 November 2012.

8. Ottoni C, Martinez-Labarga C, Vitelli L, Scano G, Fabrini E, et al. (2009) Human mitochondrial DNA variation in Southern Italy. Ann Hum Biol 36: 785–811. Available: http://www.ncbi.nlm.nih.gov/pubmed/19852679. Accessed 28 November 2012.

9. Karachanak S, Carossa V, Nesheva D, Olivieri A, Pala M, et al. (2012) Bulgarians vs the other European populations: a mitochondrial DNA perspective. International journal of legal medicine 126: 497–503. Available: http://www.ncbi.nlm.nih.gov/pubmed/21674295. Accessed 17 December 2013.

10. Terreros MC, Rowold DJ, Mirabal S, Herrera RJ (2011) Mitochondrial DNA and Y-chromosomal stratification in Iran: relationship between Iran and the Arabian Peninsula. J Hum Genet 56: 235–246. Available: http://www.ncbi.nlm.nih.gov/pubmed/21326310. Accessed 28 November 2012.

11. Battaglia V, Fornarino S, Al-Zahery N, Olivieri A, Pala M, et al. (2009) Y-chromosomal evidence of the cultural diffusion of agriculture in Southeast Europe. Eur J Hum Genet 17: 820–830. Available: http://www.ncbi.nlm.nih.gov/entrez/query.fcgi?cmd=Retrieve&db=PubMed&dopt=Citation&list_uids=19107149.

12. Pericic M, Lauc LB, Klaric IM, Rootsi S, Janicijevic B, et al. (2005) High-Resolution Phylogenetic Analysis of Southeastern Europe (SEE) Traces Major Episodes of Paternal Gene Flow Among Slavic Populations. Mol Biol Evol 22: 1964–1975. Available: http://www.ncbi.nlm.nih.gov/entrez/query.fcgi?cmd=Retrieve&db=PubMed&dopt=Citation&list_uids=15944443.

13. Erhart D, Berger B, Niederstätter H, Gassner C, Schennach H, et al. (2012) Frequency data for 17 Y-chromosomal STRs and 19 Y-chromosomal SNPs in the Tyrolean district of Reutte, Austria. International journal of legal medicine 126: 977–978. Available: http://www.ncbi.nlm.nih.gov/pubmed/22899354. Accessed 11 July 2013.

14. Petrejcíková E, Soták M, Bernasovská J, Bernasovský I, Sovicová A, et al. (2010) The genetic structure of the Slovak population revealed by Y-chromosome polymorphisms. Anthropol Sci 118: 23–30. Available: http://dx.doi.org/10.1537/ase.090203.

15. Karachanak S, Grugni V, Fornarino S, Nesheva D, Al-Zahery N, et al. (2013) Y-chromosome diversity in modern Bulgarians: new clues about their ancestry. PloS one 8: e56779. Available: http://www.pubmedcentral.nih.gov/articlerender.fcgi?artid=3590186&tool=pmcentrez&rendertype=abstract. Accessed 16 December 2013.

16. Regueiro M, Cadenas AM, Gayden T, Underhill PA, Herrera RJ (2006) Iran: tricontinental nexus for Y-chromosome driven migration. Hum Hered 61: 132–143. Available: http://www.ncbi.nlm.nih.gov/pubmed/16770078. Accessed 28 November 2012.

17. Van Oven M, Kayser M (2009) Updated comprehensive phylogenetic tree of global human mitochondrial DNA variation. Hum Mutat 30: 386–394. Available: http://www.ncbi.nlm.nih.gov/entrez/query.fcgi?cmd=Retrieve&db=PubMed&dopt=Citation&list_uids=18853457.

18. Torroni A, Huoponen K, Francalacci P, Petrozzi M, Morelli L, et al. (1996) Classification of European mtDNAs from an analysis of three European populations. Genetics 144: 1835–1850.

19. Richards MB, Macaulay VA, Bandelt H-J, Sykes BC (1998) Phylogeography of mitochondrial DNA in western Europe. Ann Hum Genet 62: 241–260. doi:10.1046/j.1469-1809.1998.6230241.x.

20. Richards M, Macaulay V, Hickey E, Vega E, Sykes B, et al. (2000) Tracing European founder lineages in the Near Eastern mtDNA pool. Am J Hum Genet 67: 1251–1276. Available: http://www.pubmedcentral.nih.gov/articlerender.fcgi?artid=1288566&tool=pmcentrez&rendertype=abstract. Accessed 25 June 2013.

21. Macaulay VA, Richards MB, Hickey E, Vega E, Cruciani F, et al. (1999) The emerging tree of West Eurasian mtDNAs: a synthesis of control-region sequences and RFLPs. Am J Hum Genet 64: 232–249. doi:10.1086/302204.

22. Rieder MJ, Taylor SL, Tobe VO, Nickerson DA (1998) Automating the identification of DNA variations using quality-based fluorescence re-sequencing: analysis of the human mitochondrial genome. Nucleic Acids Res 26: 967–973. doi:10.1093/nar/26.4.967.

23. Bandelt H-J, Forster P, Sykes BC, Richards MB (1995) Mitochondrial portraits of human populations using median networks. Genetics 141: 743–753.

24. Bandelt H-J, Forster P, Röhl A (1999) Median-joining networks for inferring intraspecific phylogenies. Mol Biol Evol 16: 37–48. doi:10.1093/oxfordjournals.molbev.a026036.

25. Hasegawa M, Di Rienzo A, Kocher TD, Wilson AC (1993) Toward a more accurate time scale for the human mitochondrial DNA tree. J Mol Evol 37: 347–354. doi:10.1007/BF00178865.

26. Allard MW, Miller K, Wilson M, Monson K, Budowle B (2002) Characterization of the Caucasian haplogroups present in the SWGDAM forensic mtDNA dataset for 1771 human control region sequences. Scientific Working Group on DNA Analysis Methods. J Forensic Sci 47: 1215–1223.

27. Soares P, Ermini L, Thomson N, Mormina M, Rito T, et al. (2009) Correcting for purifying selection: an improved human mitochondrial molecular clock. Am J Hum Genet 84: 740–759. Available: http://www.ncbi.nlm.nih.gov/entrez/query.fcgi?cmd=Retrieve&db=PubMed&dopt=Citation&list_uids=19500773.

28. Battaglia V, Fornarino S, Al-Zahery N, Olivieri A, Pala M, et al. (2009) Y-chromosomal evidence of the cultural diffusion of agriculture in Southeast Europe. Eur J Hum Genet 17: 820–830. Available: http://www.pubmedcentral.nih.gov/articlerender.fcgi?artid=2947100&tool=pmcentrez&rendertype=abstract. Accessed 26 November 2012.

29. Karafet TM, Mendez FL, Meilerman MB, Underhill PA, Zegura SL, et al. (2008) New binary polymorphisms reshape and increase resolution of the human Y chromosomal haplogroup tree. Genome Res 18: 830–838. Available: http://www.ncbi.nlm.nih.gov/entrez/query.fcgi?cmd=Retrieve&db=PubMed&dopt=Citation&list_uids=18385274.

30. Slatkin M (1995) A measure of population subdivision based on microsatelite allele frequencies. Genetics 139: 457–462.

31. Ersts P (2011) Geographic Distance Matrix Generator. Available: http://biodiversityinformatics.amnh.org/open_source/gdmg. Accessed 15 February 2013.

32. Forster P, Harding R, Torroni A, Bandelt H-J (1996) Origin and evolution of Native American mtDNA variation: a reappraisal. Am J Hum Genet 59: 935–945.

33. Saillard J, Forster P, Lynnerup N, Bandelt H-J, Nųrby S (2000) mtDNA variation among Greenland Eskimos: the edge of the Beringian expansion. Am J Hum Genet 67: 718–726.
