# Supplementary material for: Standing at the Gateway to Europe - The Genetic Structure of Western Balkan Populations Based on Autosomal and Haploid Markers
Source: PLoS One. 2014 Aug 22;9(8):e105090. doi: 10.1371/journal.pone.0105090 (PMC4141785; doi:10.1371/journal.pone.0105090)
Supplement: Text S2 — Results of the analyses of mtDNA and NRY variation. (DOC) [file pone.0105090.s033.doc]

**Supplementary Text 2. Results of the analyses of mtDNA and NRY variation.**

**MtDNA variation of studied Western Balkan populations**

***Phylogenetic analysis of mtDNA lineages of the Western Balkan region***

To improve the phylogenetic resolution of the mtDNAs of the Western Balkan region, we have performed the high resolution genotyping of all identified lineages, majority of which [98,3%; with 95% credible region (CR) width 95,1 – 99,4%)] belonged into the common western Eurasian mtDNA pool (Figures S14-18, Table S5). The data of the variation of mtDNA and NRY of Montenegrins has not been characterized before.

Haplogroup (hg) H is the most frequent and diverse hg in all analyzed Western Balkan populations. Its frequency [38,9% (CR 31,9 – 46,3%) of total sample] corresponds well with the overall frequency of hg H in most of the Europe [1–4]. The samples investigated in this study belong to 13 subclades of hg H (Table S5). The phylogenetic network of hg H lineages is presented in Figure S14A and B. Hg H1, one of the largest sub-branches of hg H, comprises about 30% of hg H lineages and 13% of the total European mtDNA pool [5]. In our sample these values were 28% (CR 18,7 – 39,6%) and 11% (CR 7,1 – 16,3%), respectively. From its seven detected sub-hgs, H1b and H1c were shared by different populations, the rest of the sub-hgs were observed only in one population sample (Table S5; Figure S14A).The frequency of other subhgs of hg H (Figure S14B) was mostly significantly lower. Hg H5 is the next largest sub-hg of H after H1 [4,6% (CR 2,4 – 8,8%)], followed by H3 and H11 [2,3% (CR 0,9 – 5,7%)] and H2a, H6, H7 and H13 [1,7% (CR 0,6 – 4,9%)]. The presence of several sub-hgs (e. g. H1s, H1u, H1q, H1e, H27, H34,H41, H55, H85) have been reported here for the first time for Balkan region, due to the shallower level of phylogenetic resolution of previous studies [6,7].

The second largest subset of maternal lineages belong to hg U (19,4%). Hg U lineages detected in this study belong to subhgs U1, U2, U4, U5 and K1 (Figure S15; Table S5), the last one of those covering more than one third of hg U lineages (6,9% from the total mtDNA pool, 36,4% from hg U).

Hg J is one of the most frequent and variable hgs in a study area (Figure S16). It comprises 11% of the mtDNA variation in Europe, with the highest frequency of 14,1% in the East Mediterranean [1] and close to that also in our sample (13,7%). In the study of Malyarchuk et al. [6] the frequency of hg J among Bosnians was 7,0%. The sub-hg J1, with defining mutations at nucleotide positions (nps) 462-3010, is the most frequent and widespread in all studied Western Balkan populations. Its sub-clade J1c is shared by all analyzed populations and is also the most frequent branch of J as well as of J1 (8,6% from total sample size ). From J1c lineages, J1c2* is the most frequent (4%from total, 29,2% from J samples) and widespread (Table S5, Figure S16).The overall frequency of hg T is half of that of its sister-clade J (5,7%) in a whole Western Balkan area (Table S5).

The phylogenetic relationships of a minor subset of mtDNA lineages of the study area that belong to macro-hg N – lineages from hgs N1 and W are depicted in Figure S17. These hgs together with hg X do have only small contribution, usually less than 5%, to the present European gene pool, although they have a wide spread area [8–12]. Note, that hg X samples of the individuals BHB5, BHB13 and BHB15 of our dataset (Table S5) have been completely sequenced within the study of Sarac et al. 2014 [13] and are available in GenBank under accession numbers KF584056, KF584057 and KF584058.

The network of phylogenetically close hgs R0a, HV and V from the macro–hg R0 is shown in Figure S18. Hg R0a is very rare in Europe. In general, its frequency is only 1,5% in the east Mediterranean and in South European populations, but it is the most frequent hg in Arabia [14].

***The results of complete sequences of mtDNA lineages of the Western Balkan region***

One of our goals was to improve the phylogenetic resolution of the hgs of uniparental markers spread in this region. We have completely sequenced 7 mtDNAs from the minor region-specific twigs of the global mtDNA tree - N1a (1), K1a (4) and R0 (2) (Figures S19-21) to better characterize the diverse gene pool of maternal lineages in the area of mixed gene flows between the Middle East and Europe.

Phylogenetic reconstruction of K1a haplotypes reveals a star-like expansion of this clade (Figure S15). Three Western Balkan samples of K1a from our dataset - Bosnian Croat, Herzegovina Croat and Serbian - have a defining mutation for sub-hg K1a13 from A to G at np 9545. These two Croat samples have also an additional transition from G to A at np 9548. To further refine the phylogenetic structure of this lineage we performed a complete sequencing of these two hg K1 genomes (Figure S19) and found the transition from T to C at np 8870, which is shared with 2 GenBank K1a13 sequences and belong to a clade K1a13a [15]. We found the same mutation from six out of 253 K1a mtDNAs from the DNA sample collection of Estonian Biocentre, all originating from Croatian mainland. Two of those (Croatia.m.(S)199 and Croatia.m.(S)341 in Figure S19) having different HVS-1 motifs were sequenced completely. Novel mutation G9548A together with the shared mutation C11236T with GenBank sequence (JN202723, Dr. M. van Oven personal communication) let us define a new sub-clade of K1a13a, which we propose here to be named as K1a13a1 (see also main text).

We found one N1a sample from the Western Balkan region among Croatians, which did not have diagnostic positions for N1a1a1a [15]. The transition at np 207 is observed also in other sub-branches of N1a1, but the transition at np 4838 is a novel in N1a phylogeny (Figure S20).Two complete sequences belonged into the hg R0a2, one of those into R0a2c (Figure S21).

***MtDNA statistics of the sample of Western Balkan region***

Kosovars and Bosnian Serbs showed the lowest values both for gene as well as for nucleotide diversity, which can be the result of a founder effect reflected by a number of shared haplotypes (Table S7). On the other side of the scale are Bosniacs, Croatians and Macedonians who have the highest value of gene diversity. Nucleotide diversity is highest among Montenegrins.

All ethnic groups living in Bosnia and Herzegovina - Bosniacs, Bosnian Croats and Bosnian Serbs - showed close maternal affinities. The major proportion of genetic variation was observed inside the ethnic groups (0,41%), the variation between the groups was much lower (0,13%, Table S9). It is expected, because these populations are supposed to be highly admixed both in Bosnia and Herzegovina as well as in a whole studied Western Balkan Peninsula region and are isolated from each other neither linguistically nor geographically.

The variation within populations is consistent with previous studies on mtDNA worldwide [1]. The results of AMOVA analysis for three religion groups (Table S9) gave higher values for differences among group (0,34%) than those for groupings of geographic and linguistic affiliations, but it is still not very low. Differences among populations within groups encompass 0,29% of a variation and the variation within populations is 99,38%. In order not to lose the phylogenetic affiliations of the maternal lineages we made a correlation analysis also with Mantel test by the use of the data of mtDNA hg frequencies. These results showed that the variation of mtDNA correlates significantly neither with spatial distribution nor with linguistic affiliations of analyzed populations (Table S9).

***PCA analysis of haploid markers of Western Balkan populations***

PCA analyses in Figure S13 sum up the variation of haploid markers. Due to the small size of the analyzed NRY sample (Table S6), the variation of paternal lineages of the Western Balkan region was done by the use of a pooled dataset of our and published data. Closest to the Western Balkan populations in the European context are the Slavic-speaking Belarusians and Czechs in case of mtDNA (Figure S13A, C) and Czechs and Slovaks in case of NRY (Figure S13B, D).

If to look at the distribution of paternal lineages inside the Western Balkan cluster, the variation of Bosnian Serbs and Bosniacs follows the same pattern and these populations cluster closely together. In addition, the NRY lineages of Bosnian Croats are closer to other ethnic groups of Bosnia and Herzegovina than to Croatians (Figure S13D).

The differences of Kosovars from other Western Balkan populations seen in autosomal analyses (Figures 3, 5, Table S3) are not so obvious at the NRY or mtDNA level (Figures S13C, D). Like in case of autosomal profiles (Figure 3, 5, S2), the PCA of paternal lineages puts Kosovars close to Macedonians. Although the variation of their maternal lineages seats them separately from the rest of the studied populations (Figure S13C), the large deviations inside the Western Balkan cluster due to the small sample sizes of different ethnic groups does not let us decide about the similarities of differences between the members of this cluster.

**References**

1. Richards M, Macaulay V, Hickey E, Vega E, Sykes B, et al. (2000) Tracing European founder lineages in the Near Eastern mtDNA pool. Am J Hum Genet 67: 1251–1276.

2. Achilli A, Rengo C, Magri C, Battaglia V, Olivieri A, et al. (2004) The molecular dissection of mtDNA haplogroup H confirms that the Franco-Cantabrian glacial refuge was a major source for the European gene pool. Am J Hum Genet 75: 910–918. doi:10.1086/425590.

3. Loogväli E-L, Roostalu U, Malyarchuk BA, Derenko M V, Kivisild T, et al. (2004) Disuniting uniformity: a pied cladistic canvas of mtDNA haplogroup H in Eurasia. Mol Biol Evol 21: 2012–2021. doi:10.1093/molbev/msh209.

4. Roostalu U, Kutuev I, Loogvali E-L, Metspalu E, Tambets K, et al. (2007) Origin and Expansion of Haplogroup H, the Dominant Human Mitochondrial DNA Lineage in West Eurasia: The Near Eastern and Caucasian Perspective. Mol Biol Evol 24: 436–448. doi:10.1093/molbev/msl173.

5. Pereira L, Richards M, Alonso A, Albarrán C, Garcia O, et al. (2004) Subdividing mtDNA haplogroup H based on coding-region polymorphisms—a study in Iberia. Int Congr Ser 1261: 416–418. doi:10.1016/S0531-5131(03)01651-0.

6. Malyarchuk BA, Grzybowski T, Derenko M V, Czarny J, Drobnic K, et al. (2003) Mitochondrial DNA variability in Bosnians and Slovenians. Ann Hum Genet 67: 412–425. doi:10.1046/j.1469-1809.2003.00042.x.

7. Cvjetan S, Tolk HV, Lauc LB, Colak I, Dordević, et al. (2004) Frequencies of mtDNA haplogroups in southeastern Europe--Croatians, Bosnians and Herzegovinians, Serbians, Macedonians and Macedonian Romani. Coll Antropol 28: 193–198.

8. Finnilä S, Lehtonen MS, Majamaa K (2001) Phylogenetic network for European mtDNA. Am J Hum Genet 68: 1475–1484. doi:10.1086/320591.

9. Malhi RS, Schultz BA, Smith DG (2001) Distribution of mitochondrial DNA lineages among Native American tribes of Northeastern North America. Hum Biol 73: 17–55. doi:10.1353/hub.2001.0008.

10. Reidla M, Kivisild T, Metspalu E, Kaldma K, Tambets K, et al. (2003) Origin and Diffusion of mtDNA Haplogroup X. Am J Hum Genet 73: 1178–1190. doi:10.1086/379380.

11. Metspalu M, Kivisild T, Metspalu E, Parik J, Hudjashov G, et al. (2004) Most of the extant mtDNA boundaries in South and Southwest Asia were likely shaped during the initial settlement of Eurasia by anatomically modern humans. BMC Genet 5: 26. doi:10.1186/1471-2156-5-26.

12. Derenko M, Malyarchuk B, Grzybowski T, Denisova G, Dambueva I, et al. (2007) Phylogeographic analysis of mitochondrial DNA in northern Asian Populations. Am J Hum Genet 81: 1025–1041. doi:10.1086/522933.

13. Sarac J, Sarić T, Auguštin DH, Jeran N, Kovačević L, et al. (2014) Maternal genetic heritage of Southeastern Europe reveals a new Croatian isolate and a novel, local sub-branching in the x2 haplogroup. Annals of human genetics 78: 178–194. doi:10.1111/ahg.12056.

14. Cerný V, Mulligan CJ, Fernandes V, Silva NM, Alshamali F, et al. (2011) Internal diversification of mitochondrial haplogroup R0a reveals post-last glacial maximum demographic expansions in South Arabia. Mol Biol Evol 28: 71–78. doi:10.1093/molbev/msq178.

15. Van Oven M, Kayser M (2009) Updated comprehensive phylogenetic tree of global human mitochondrial DNA variation. Hum Mutat 30: 386–394. doi:10.1002/humu.20921.
